# Particle-associated N_2_ fixation by heterotrophic bacteria in the global ocean

**DOI:** 10.1126/sciadv.adq4693

**Published:** 2025-02-19

**Authors:** Subhendu Chakraborty, Ken H. Andersen, Agostino Merico, Lasse Riemann

**Affiliations:** ^1^ Systems Ecology Group, Leibniz Centre for Tropical Marine Research (ZMT), Bremen, Germany.; ^2^ Department of Biology, Marine Biological Section, University of Copenhagen, Helsingør, Denmark.; ^3^ Centre for Ocean Life, DTU Aqua, Technical University of Denmark, Kemitorvet, Kgs. Lyngby, Denmark.; ^4^ Faculty of Biology and Chemistry (FB2), University of Bremen, Bremen, Germany.

## Abstract

N_2_-fixing microorganisms (diazotrophs) sustain life on our planet by providing biologically available nitrogen to plants. In the oceans, cyanobacterial diazotrophs, mostly prevalent in warm tropical and subtropical waters, were traditionally considered the sole contributors to marine N_2_ fixation. Recently, an almost ubiquitous distribution of N_2_-fixing heterotrophic bacteria has been discovered in the pelagic ocean. However, the mechanisms enabling heterotrophic diazotrophs to thrive in cold high-latitude waters and their contribution to the global nitrogen budget are unknown. Using a data-driven cell-based metabolic model, we show that heterotrophic bacteria inside sinking particles can fix N_2_ over a wide range of temperatures, explaining their ubiquitous presence in the oceans. We estimate that heterotrophic diazotrophs account for about 10% of global marine N_2_ fixation, with the highest contribution in oxygen minimum zones. These findings call for a reassessment of the N_2_ fixation patterns and the biogeochemical cycling of nitrogen in the global ocean.

## Teaser

Heterotrophic bacteria fix N_2_ in more diverse environments than previously thought, critical for the oceanic nitrogen cycle.

## INTRODUCTION

Biological nitrogen (N_2_) fixation, the conversion of inert N_2_ gas into ammonia by specialized prokaryotes (diazotrophs), is a critically important source of bioavailable nitrogen and controls primary productivity in oligotrophic oceans (*1*). Until recently, N_2_ fixation in marine waters was thought to be almost exclusively carried out by cyanobacterial diazotrophs in tropical and subtropical surface waters. Accumulating evidence, however, suggests that noncyanobacterial diazotrophs, especially N_2_-fixing heterotrophic bacteria, are widespread and actively fix N_2_ in marine waters, from the tropics to the poles and from the surface to the abyss (*2*–*4*). Given that N_2_ fixation is an anaerobic process and that cellular protection from O_2_ is energetically costly (*5*), N_2_ fixation by heterotrophic diazotrophs in oxygenated ocean waters (*6*) is somewhat paradoxical. The availability of dissolved organic matter is generally sparse and limits the growth of free-living heterotrophic diazotrophs (*3*, *4*). Sinking marine particles, being rich in organic matter and poor in O_2,_ could be suitable loci for heterotrophic diazotrophs (*7*). This possibility is supported by the fact that marine heterotrophic diazotrophs are generally chemotactic (*8*) and able to efficiently colonize surfaces (*9*, *10*) and particulate matter (*11*, *12*) and that, recently, N_2_ fixation by heterotrophic diazotrophs was documented on sinking particles (*12*, *13*). However, the mechanisms that allow particle-associated heterotrophic diazotrophs to fix N_2_ under a broad range of environmental conditions, including cold, high-latitude waters, are unknown.

Nitrogenase genes (*nifH*) and transcripts from heterotrophic diazotrophs are almost ubiquitously detected throughout the oceans (*2*, *4*, *6*). The relative abundance of polymerase chain reaction (PCR)–amplified *nifH* genes of heterotrophic diazotrophs often exceeds that of their cyanobacterial counterparts (*2*, *3*). However, gene-based information does not accurately reflect actual N_2_ fixation rates. Laboratory measurements of cellular level N_2_ fixation by heterotrophic diazotrophs are scarce due to the lack of culture representatives, but a few exist (*10*, *14*–*16*). Also, only a few direct measurements of community N_2_ fixation rates exist from locations where cyanobacteria were reported to be absent (*6*). Moreover, conceivably due to the low rates and associated methodological challenges, only a few studies have documented in situ N_2_ fixation by heterotrophic diazotrophs associated with sinking particles. For example, active N_2_ fixation by heterotrophic diazotrophs was detected in particle enrichment incubations where cyanobacterial photosynthesis was inhibited (*17*). Another recent study reported cell-specific heterotrophic N_2_ fixation rates measured in situ, using particle-targeted nanoscale secondary ion mass spectrometry (nanoSIMS), in pelagic waters of the North Pacific (*13*). Given this paucity of data, the contribution of particle-associated heterotrophic diazotrophs to the global nitrogen budget and their importance in marine nitrogen biogeochemistry remain enigmatic.

N_2_ fixation rates inside sinking particles are likely affected by the particle size, which can vary from micrometers to several millimeters (*18*). The particle size spectrum follows a power law relationship with a reduction in particle abundance as size increases (*19*). Because of smaller surface-to-volume ratios, large particles are more prone to develop an anoxic interior, which enhances the possibility of N_2_ fixation. However, the exponent of the power law, which determines the relative abundance of small to large particles, varies substantially throughout the global ocean (*20*). Moreover, by regulating the sinking speed, particle size influences how quickly particles descend through depths of varying O_2_ concentrations, affecting (i) the extent to which an anoxic interior develops and (ii) the particle-associated heterotrophic N_2_ fixation. Although the particle size can have these variable impacts, it is unclear how it affects, in combination with environmental conditions, heterotrophic N_2_ fixation in the global ocean.

**Fig. 1.**
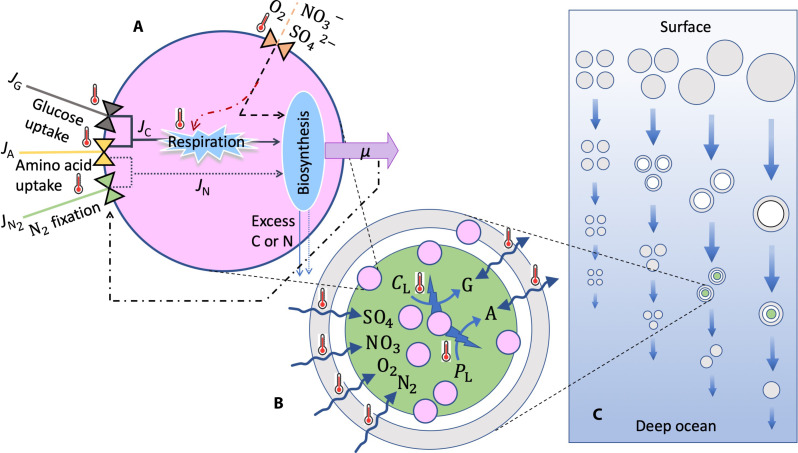
Schematic representation of different components of the dynamic model. (**A**) Dynamics inside a single cell: Fluxes of carbon (*J*_C_; solid lines), nitrogen (*J*_N_; dotted lines), and electron acceptors (O_2_, NO_3_^−^, and SO_4_^2−^; dashed line) are combined (blue ellipse) to drive cell division rate (μ; magenta arrow) after respiratory costs are accounted for (blue explosion). Triangles indicate the processes underpinning the uptake of glucose (*J*_G_), amino acids (*J*_A_), N_2_ fixation ($J_{N_{2}}$), and diffusive inflow of O_2_, NO_3_^−^, and SO_4_^2−^. The excess amounts of assimilated C or N are excreted from the cell (thin blue arrows). The red dashed-dot line represents the regulation of respiration by electron acceptors. Temperature-dependent processes are indicated with red thermometer symbols. (**B**) Dynamics inside a single particle: The particle interior has three distinct zones: O_2_ present (gray), O_2_ absent without N_2_ fixation (white), and O_2_ absent with N_2_ fixation (green). The exact sizes of the zones are emergent outcomes of the model. Bacterial cells (pink circles) hydrolyze polysaccharides (*C*_L_) and polypeptides (*P*_L_) into glucose (*G*) and amino acids (*A*), respectively. Diffusive exchanges of N_2_, O_2_, NO_3_, SO_4_, glucose, and amino acids between the particle interior and surrounding water depend on the concentration gradients. (**C**) Dynamics in the water column: Particles of different sizes (range of radiuses: 5 μm to 0.25 cm) sink through the water column starting from the surface ocean toward the bottom while being reduced in size as they are being remineralized by the bacteria. The figure is inspired by Bianchi *et al.* (*85*).

Here, we use a data-informed mathematical model of cell physiology and biogeochemistry that represents facultative N_2_-fixing heterotrophic bacteria living inside sinking marine particles and incorporates the temperature dependence of cellular and particle processes relevant to N_2_ fixation. Starting from basic physiological processes determining growth and N_2_ fixation in an individual bacterial cell, we allow cells to grow in sinking particles in a water column (Fig. 1). We then upscale the model to the global ocean to explain how cellular mechanisms determine the global distribution of N_2_ fixation by particle-associated heterotrophic diazotrophs. The core of the model is a description of a general heterotrophic bacterial cell in which the cellular N_2_ fixation rate is not prescribed a priori but is an emergent property depending on cellular and surrounding environmental conditions. We impose strong constraints on the model by considering parameter values from experiments, observations, and literature sources and by running global simulations using observed vertical gradients of temperature, O_2_, and NO_3_^−^ concentrations (*21*–*23*). We specifically examine (i) the mechanisms that allow heterotrophic diazotrophs associated with sinking particles to survive and fix N_2_ over a broad range of temperatures, (ii) the concurrence of a variety of environmental conditions regulating N_2_ fixation by heterotrophic diazotrophs in different parts of the global ocean, (iii) the global distribution of N_2_ fixation by heterotrophic diazotrophs, and (iv) the contribution of heterotrophic diazotrophs to the global N_2_ fixation.

## RESULTS

### Overview of the model

The model represents a population of facultative N_2_-fixing heterotrophic bacteria that live inside sinking particles and regulate N_2_ fixation to meet basic needs for growth. It incorporates temperature regulation of cellular and particle processes and, when embedded in the global ocean, demonstrates how fundamental cellular mechanisms determine the global distribution of N_2_ fixation by heterotrophic bacteria associated with sinking particles. The model consists of two parts: a “cell model” and a “particle model.” The particle model is then embedded in a water column and lastly extended to the global ocean. A schematic representation of the model is given in Fig. 1, a full description of the model is provided in Materials and Methods, while the details and values of parameters and environmental variables are available in the Supplementary Materials.

The cell model describes the basic cellular processes of an individual heterotrophic bacterium: resource uptake (glucose and amino acids), uptake of electron acceptors (O_2_, NO_3_^−^, and SO_4_^2−^), respiration, and cell division (Fig. 1A). The cell primarily uses O_2_ to maintain respiration. However, it can also use NO_3_^−^ or even SO_4_^2−^ for respiration when, respectively, O_2_ or both O_2_ and NO_3_^−^ are unavailable. The cell fixes N_2_ when the nitrogen demand cannot be met by the amount of organic nitrogen available inside the particle. Since nitrogenase is irreversibly inhibited by O_2_, one of the main prerequisites for N_2_ fixation is to keep the cell O_2_ free. We assume that the cell can increase respiration to burn the cellular O_2_ in excess (*24*). Therefore, the process of O_2_ removal requires a sufficient amount of supply of carbon. Temperature dependencies are implemented on the specific cellular processes using Q_10_ factors. The cell also regulates the rate of N_2_ fixation to maximize its division rate μ*. The overall cellular N_2_ fixation rate is therefore an emergent property of the model and depends on the availability of carbon, nitrogen, cellular O_2_ concentration, and temperature.

The cell model is embedded in a particle model, which describes the interactions of cells with their immediate environment inside particles (Fig. 1B). Bacteria colonize particles and use ectoenzymes to degrade the labile part of polymers (polysaccharides and polypeptides) to oligomers or monomers (glucose and amino acids), which can be efficiently taken up by bacteria. Note that, when the labile components are exhausted, the particle is left with only the nonlabile components, which are inaccessible to bacteria. The diffusive exchanges of glucose, amino acids, O_2_, and NO_3_^−^ depend on the concentration gradient between the particle interior and the surrounding environment. Because of the high concentrations of SO_4_^2−^ (28 mM) (*25*) and N_2_ (0.4 mM) (*26*) in seawater, the uptakes of these two components are assumed to be limited by the cellular maximum uptake capacities and not by the rate of diffusion into the cell. The temperature dependency of diffusivity is based on the Walden’s rule (*27*) and the temperature-dependent viscosity of water (*28*).

We assume that the particle size spectrum in the upper ocean follows a power law (*19*), and the sinking speed of particles depends on particle size (Fig. 1C) (*29*). While sinking through the water column, particles face gradients of temperature, O_2_, and NO_3_^−^ concentrations, and bacterial degradation reduces their size and the content of labile polymers.

The model of sinking particles is then run in the global ocean at every 5° by 5° grid point using a space-resolved particle distribution at the ocean surface (*30*) and vertical fields of annual mean temperature, O_2_, and NO_3_^−^ concentrations from the World Ocean Atlas (*21*–*23*).

### Thermal range of N_2_ fixation by heterotrophic bacteria inside a particle

**Fig. 2.**
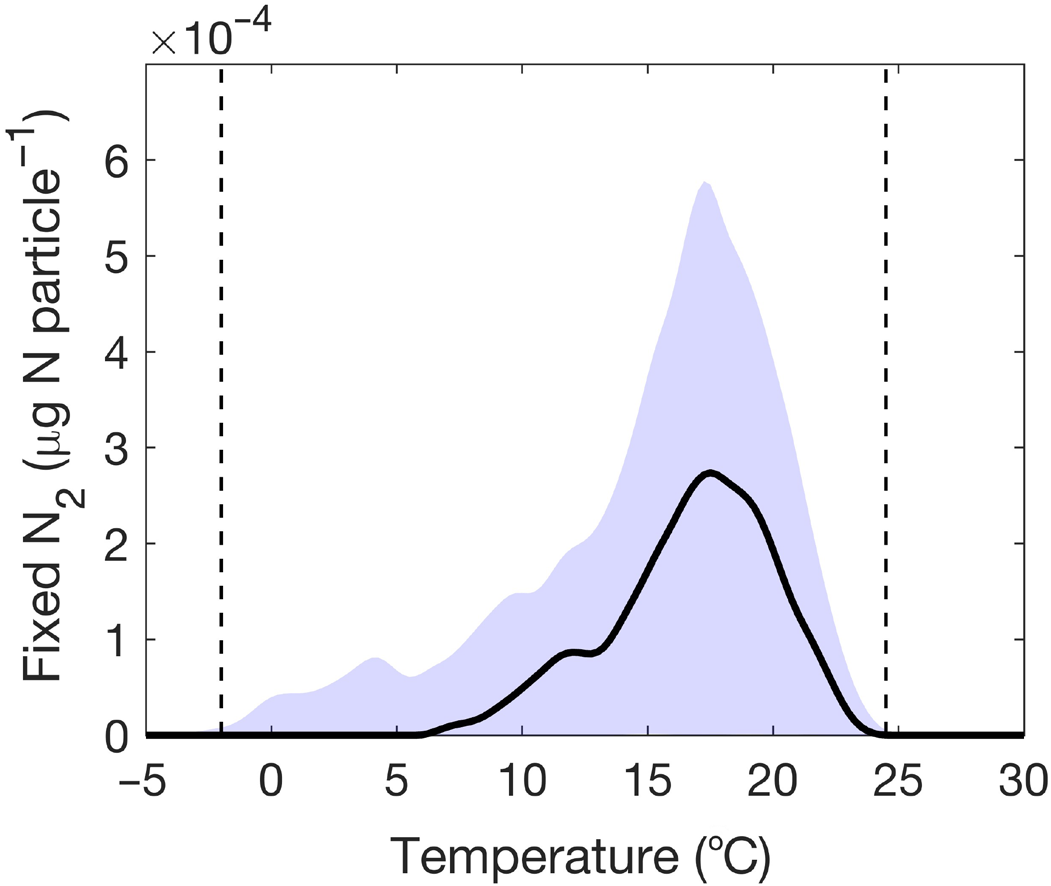
Thermal range of N_2_ fixation by particle-associated heterotrophic bacteria. N_2_ fixation (black solid line) is estimated at different temperatures in a particle of radius 0.15 cm with O_2_ and NO_3_^−^ concentrations of 200 and 10 μM, respectively, and initial concentrations of labile polysaccharides and polypeptides of, respectively, 8 × 10^7^ μg G liter^−1^ and 1.5 × 10^8^ μg A liter^−1^. The shaded area marks the range of fixed N_2_ obtained by varying (i) the initial concentrations of polysaccharides and polypeptides and (ii) the *Q*_10_ values related to hydrolysis and uptakes of glucose and amino acids by ±25% from nominal values. The vertical dashed lines mark the maximum possible thermal range of N_2_ fixation.

**Fig. 3.**
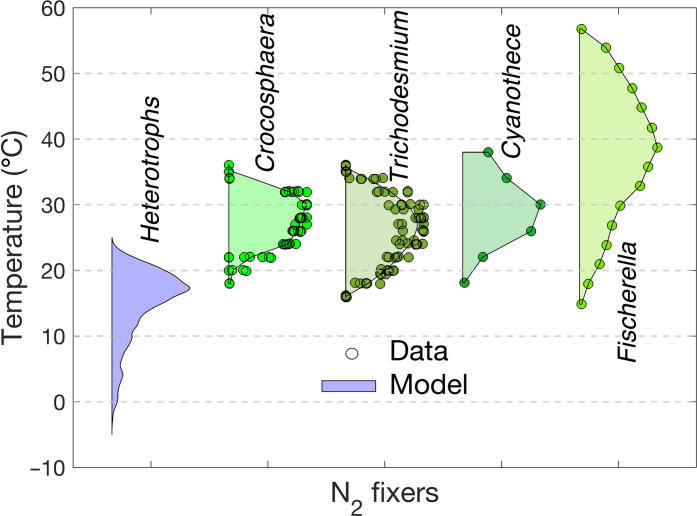
Comparison of thermal ranges of N_2_ fixation activity. The range of temperature of N_2_ fixation estimated by our model for heterotrophic diazotrophs (blue) compared to those measured in laboratory experiments for different cyanobacteria (green shades), including *Trichodesmium* (*31*, *33*), *Crocosphaera* (*31*, *34*), *Cyanothece* (*32*), and *Fischerella* (*35*). Circles represent experimental data. N_2_ fixation rates are normalized by the corresponding organismal maximum fixation rates. The maximum range of N_2_ fixation by heterotrophic bacteria is the same as in Fig. 2.

Under fixed environmental conditions (O_2_ and NO_3_^−^), our model predicts a broad temperature range of N_2_ fixation, spanning from 6° to 24°C, with the highest N_2_ fixation rate at 17°C (Fig. 2). Our sensitivity analysis shows that N_2_ fixation can occur even under negative temperatures, down to −2°C (shaded region in Fig. 2). In comparison, the temperature range of the prominent cyanobacterial diazotrophs *Trichodesmium*, *Crocosphaera*, and *Cyanothece* is 18° to 38°C, with highest N_2_ fixation rates at 24° to 32°C (Fig. 3) (*31*–*34*). The heterocyst-forming cyanobacterium *Fischerella* sp. can fix N_2_ at an even wider range of temperatures, 15° to 57°C, with the highest nitrogenase activity at 40°C (*35*).

Within the viable temperature range, we find that the hydrolyzation of polysaccharides and polypeptides into glucose and amino acids enables heterotrophic bacteria to grow inside the particle (Supplementary Text 1 and fig. S1). Respiration rates create an anoxic interior that allows bacteria to perform N_2_ fixation when nitrogen from amino acids is exhausted. When the labile components of polysaccharides and polypeptides are exhausted, the depletion of glucose eventually terminates N_2_ fixation. However, we find that the variations in temperature-driven regulations of different cellular processes (hydrolyzation rate of polymers, resource uptake, respiration), and the diffusion of materials into the particle (glucose, amino acids, O_2_, and NO_3_) interact to determine the rate of N_2_ fixation and the thermal range of N_2_ fixation by heterotrophic diazotrophs. A detailed mechanistic explanation of the sequential processes governing N_2_ fixation within the particle at different temperatures is provided below.

**Fig. 4.**
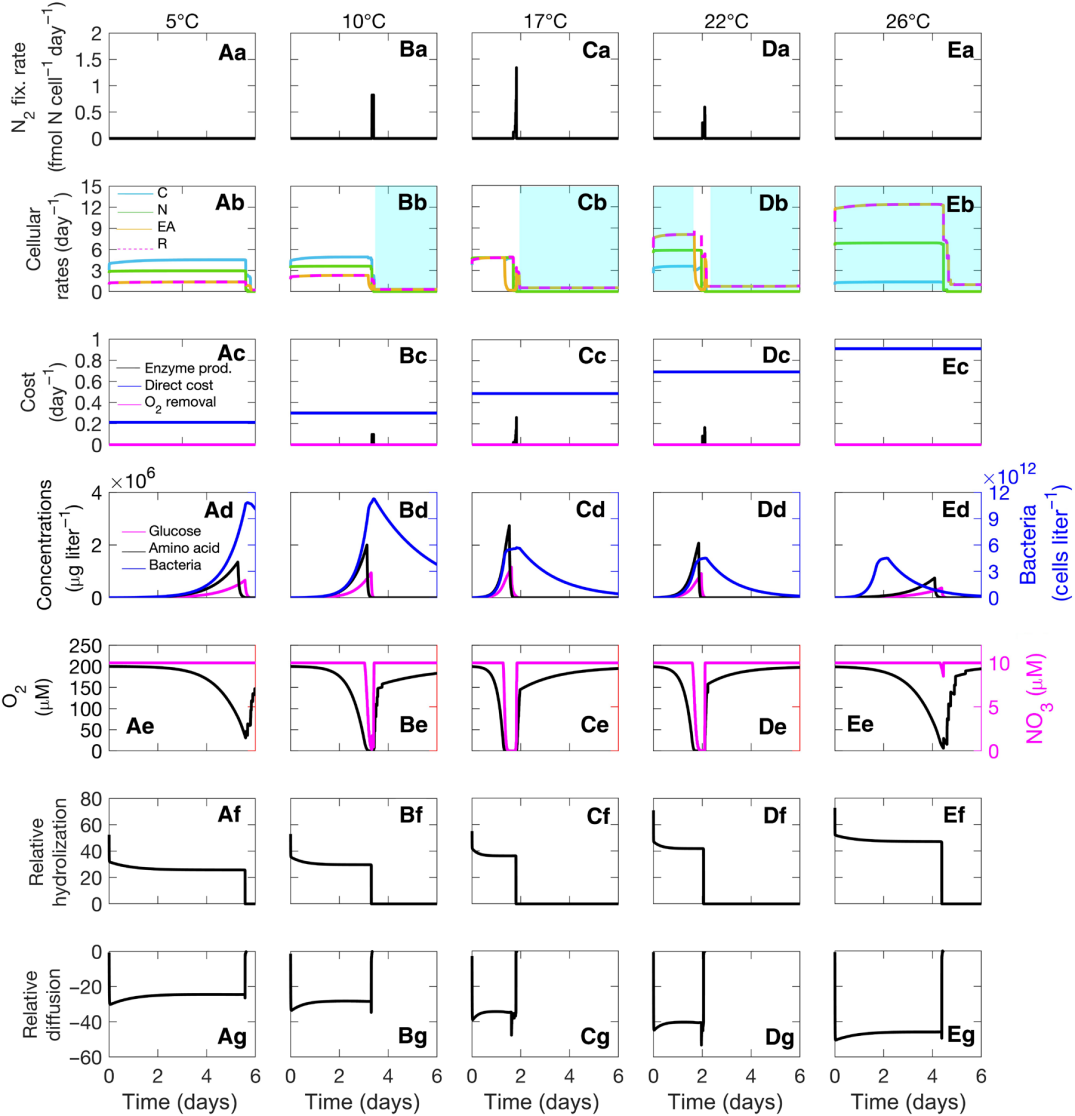
Temporal dynamics of cellular rates and concentrations. Rates and concentrations are calculated at a distance of 0.05 cm from the center of a particle of radius 0.15 cm and at different temperatures, with each column representing a specific temperature. (**Aa** to **Ea**) Cellular N_2_ fixation rates. (**Ab** to **Eb**) Rates of carbon (blue line), nitrogen (green line), and electron acceptor (orange line) available to the cell for biomass synthesis and total respiration rate (dashed magenta line). The blue-shaded region marks the period of carbon limitation. (**Ac** to **Ec**) Respiratory costs related to N_2_ fixation in terms of enzyme production (blue line), direct respiration (black line), and O_2_ removal (magenta line). (**Ad** to **Ed**) Glucose, amino acids, and bacterial concentrations in the particle. (**Ae** to **Ee**) O_2_ (black line) and NO_3_^−^ (magenta line) concentrations in the particle. (**Af** to **Ef**) The hydrolyzation rate of glucose relative to its uptake, defined by the quotient between the hydrolyzation rate and the uptake rate. The positive relative hydrolyzation is due to excess hydrolyzation compared to uptake. (**Ag** to **Eg**) The diffusion rate of glucose relative to its uptake, defined by the quotient between the diffusion rate and the uptake rate. The negative relative diffusion is due to the excess outflow of hydrolyzed materials compared to uptake. These temporal dynamics are obtained with the same environmental conditions as in Fig. 2.

1) At temperatures lower than 6°C, low rates of cellular respiration (Fig. 4Ac) fail to develop an anoxic environment inside the particle (Fig. 4Ae). Moreover, because of the low hydrolyzation rate (Fig. 4Af), the amount of available carbon is also insufficient to support increased respiration to keep the cell O_2_ free. Therefore, the cells are unable to perform N_2_ fixation (Fig. 4Aa).

2) As temperature exceeds 6°C, cellular respiration increases, resulting in the formation of an anoxic interior (Fig. 4Be), which enables bacteria to fix N_2_ (Fig. 4Ba) to support cellular growth in the absence of nitrogen from amino acids (Fig. 4Bd). However, the overall low cellular rates at relatively low temperatures slow down bacterial growth (Fig. 4Bd) and limit the capacity to create an anoxic environment, thus delaying the onset of N_2_ fixation in the particle. Consistent with our model results, a delay in the N_2_ fixation due to reduced nitrogenase activity at low temperatures was previously observed in a laboratory experiment with the unicellular cyanobacterium *Cyanothece* sp. (*32*).

3) With increasing temperature, the rate of N_2_ fixation increases. At around 17°C, conditions are optimal, and the balance between available carbon from hydrolyzation, electron acceptors through diffusion, and total respiration produces the highest N_2_ fixation rate, 2.7 × 10^−4^μg N particle^−1^ (Fig. 2 and Fig. 4Ca). Our predicted optimal temperature lies within the experimentally observed optimal temperature range, 12° to 18°C, of N_2_ fixation by heterotrophic bacteria in association with Mediterranean seagrass (*36*). The short time span (only a few hours) of inner particle anoxia produced by our model matches well with the temporal window of anoxic conditions observed using microsensors inside laboratory-made aggregates (*37*).

4) With a further increase in temperature, the N_2_ fixation rate sharply declines. We find that higher temperatures increase the hydrolyzation of polysaccharides and polypeptides (Fig. 4Df) at rates that exceed the subsequent increase in the uptake of hydrolyzed materials. Higher temperature also increases the rate of diffusive outflow of hydrolyzed materials (Fig. 4Dg), which leads to an inefficient use of glucose and amino acids. Subsequently, the availability of carbon limits cellular growth and N_2_ fixation (indicated by the blue-shaded region in Fig. 4Db).

5) Last, at temperatures higher than 24°C, the loss of hydrolyzed materials through diffusive outflow becomes so high (Fig. 4Eg) that the cell remains carbon limited (Fig. 4Eb) and unable to perform N_2_ fixation (Fig. 4Ea).

Overall, we find that low respiration rates limit N_2_ fixation at low temperatures, whereas the mismatch between the high rate of hydrolyzation and inefficient uptake of hydrolyzed materials limits N_2_ fixation at high temperatures. We also find that the initial concentration of polysaccharide regulates the amount of fixed N_2_, whereas the minimum temperature of N_2_ fixation is determined by the temperature sensitivity of resource uptakes (fig. S2). Moreover, heterotrophic N_2_ fixation inside particles is more likely to develop in particles with diameters larger than 0.06 cm (fig. S3). Our estimated cell-specific N_2_ fixation rates (up to 1.4 fmol cell^−1^ day^−1^) are comparable with N_2_ fixation rates of heterotrophic bacteria measured in laboratory experiments (0.02 to 1.1 fmol cell^−1^ day^−1^) (*10*, *15*) and in situ (0.05 to 8.61 fmol cell^−1^ day^−1^) (*13*).

### Latitudinal variation in particle-associated N_2_ fixation

**Fig. 5.**
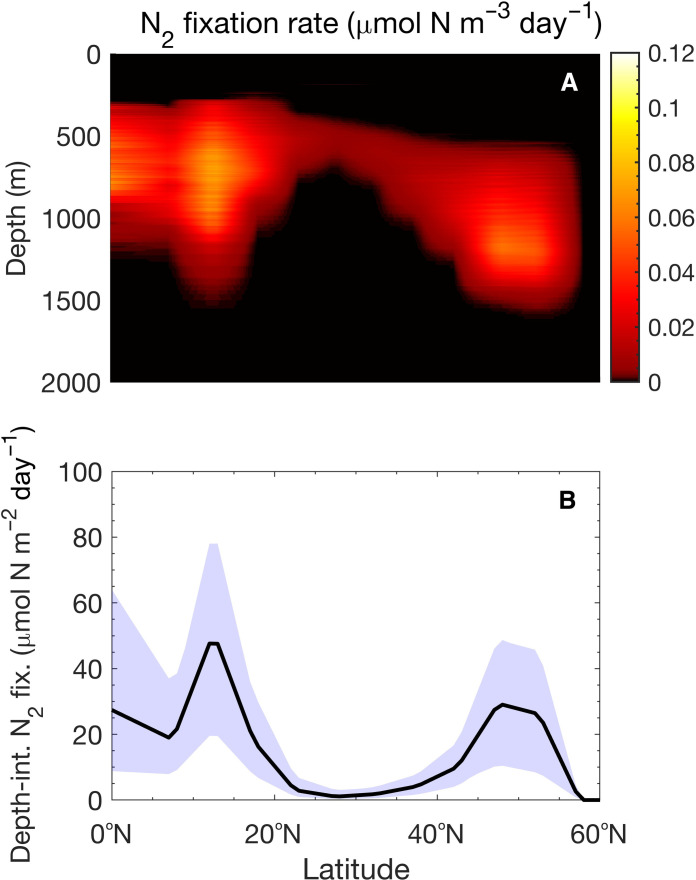
Latitudinal variation of N_2_ fixation rates by heterotrophic diazotrophs in sinking marine particles in the North Pacific (at 137.5°W). (**A**) Vertical distribution of volumetric N_2_ fixation rates at different latitudes. (**B**) Depth-integrated N_2_ fixation rates by particle-associated heterotrophic bacteria at different latitudes. The blue-shaded region marks the range of N_2_ fixation rates obtained by varying (i) the initial concentrations of polysaccharides and polypeptides, (ii) *Q*_10_ values for the hydrolysis, and (iii) *Q*_10_ values for uptake rates of glucose and amino acids by ±25% from nominal values (reported in table S1).

We further investigate the variations in N_2_ fixation by heterotrophic bacteria across latitudinal gradients, spanning from 0° to 60°N at 137.5°W, by considering (i) changes in O_2_, NO_3_^−^, and temperature (Fig. 5) in the water columns and (ii) latitude-specific particle distributions at the ocean surface. We find that particle-associated heterotrophic bacteria can fix N_2_ over a broad range of latitudes and depths (up to 1500 m). The maximum volumetric N_2_ fixation rate appears at around 750 m in low latitudes (Fig. 5A). The maximum depth-integrated N_2_ fixation rate is at around 12°N (Fig. 5B). Although low-latitude waters (lower than 22°N) emerge as hotspots, notable N_2_ fixation occurs at high latitudes (from 40° to 57°N).

**Fig. 6.**
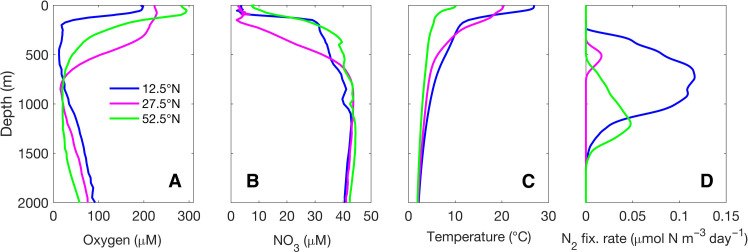
Water column N_2_ fixation rates at contrasting latitudes along 137.5°W. Vertical distribution of (**A**) O_2_, (**B**) NO_3_^−1^, (**C**) temperature, and (**D**) N_2_ fixation rates per unit volume of water at 12.5°N (blue), 27.5°N (magenta), and 52.5°N (green).

Temperature and O_2_ concentrations are the main environmental factors determining the latitudinal distribution of particle-associated N_2_ fixation (Supplementary Text 2). Water columns with low surface O_2_ concentrations (less than 200 μM) and a wide hypoxic layer, starting within the upper 250 m of the water column (Fig. 6A and fig. S3A), stimulate N_2_ fixation (Figs. 5A and 6D). In addition, high surface water temperatures (Fig. 6C and fig. S3C), which favor bacterial growth, promote N_2_ fixation in relatively shallow waters at low latitudes (Figs. 5A and 6D).

### Patterns and contribution of particle-associated N_2_ fixation in the global ocean

#### Oxygen minimum zones

**Fig. 7.**
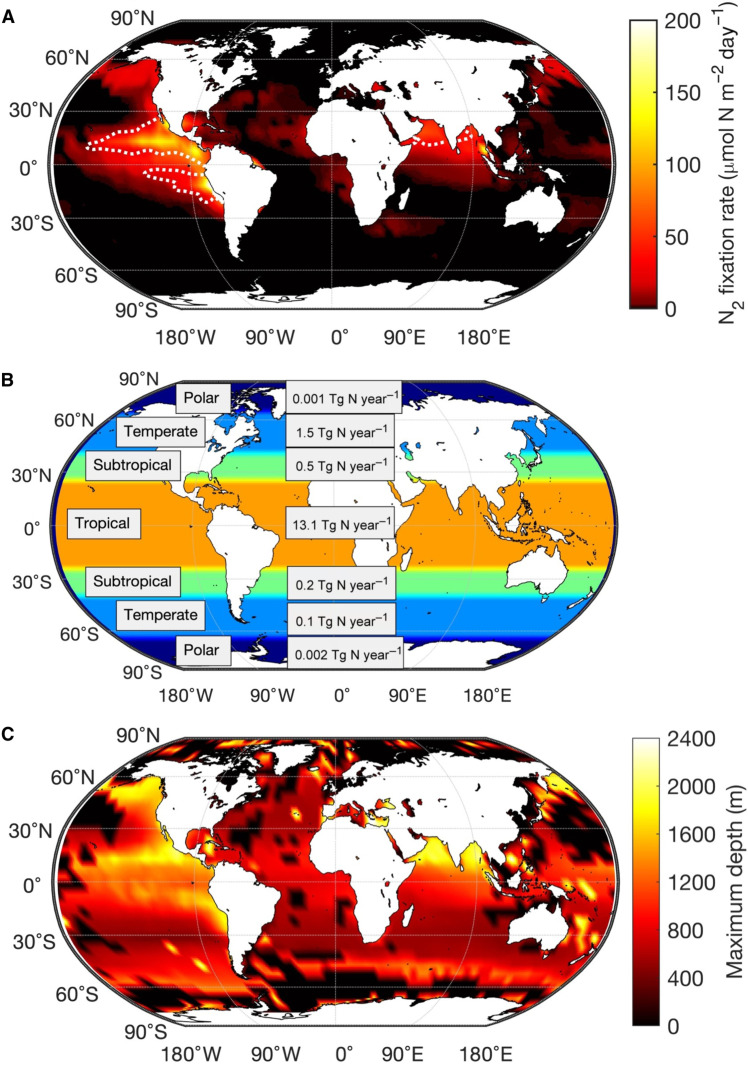
N_2_ fixation by heterotrophic diazotrophs associated with sinking particles in the global ocean. (**A**) Patterns of depth-integrated N_2_ fixation rates predicted by our model. Dotted white contours enclose suboxic (lower than 5 μmol O_2_ liter^−1^) regions. (**B**) Total N_2_ fixation rates at different regions of the globe. The oceanic regions, indicated by different colors, are partitioned into tropical, subtropical, temperate, and polar zones. (**C**) Maximum depth of occurrence of particle-associated N_2_ fixation in the global ocean.

Last, we consider (i) variations in annual mean O_2_, NO_3_^−^, and temperature in the water columns and (ii) spatially resolved annual mean particle distributions at the ocean surface in the global ocean. Under these conditions, our model predicts high rates of N_2_ fixation in the major oxygen minimum zones (OMZs): the eastern tropical South Pacific, the eastern tropical North Pacific, the Arabian Sea, and the Bay of Bengal (Fig. 7A). Our estimates show a maximum volumetric N_2_ fixation rate of 2 μmol N m^−3^ day^−1^ in the eastern tropical South Pacific around 850-m depth. At first sight, the volumetric N_2_ fixation rates appear low (fig. S4), but when integrated over the overall depth of activity in the water column, ranging from 200 to 2000 m, the rates become substantial, up to ~200 μmol N m^−2^ day^−1^. We estimate that particle-associated heterotrophic diazotrophs supply 9.4 Tg N year^−1^ of fixed N_2_ in the OMZs. Considering the previously reported global N_2_ fixation rate of 163 Tg N year^−1^ (*38*), the nitrogen fixed by particle-associated heterotrophic diazotrophs in the OMZs accounts for ~6% of the global N_2_ fixation.

#### N_2_ fixation in other parts of the global ocean

N_2_ fixation by particle-associated heterotrophic bacteria is widely distributed across the global ocean (Fig. 7A). Although the tropical oceans are hotspots, the northern temperate Pacific Ocean emerges as a very active region of N_2_ fixation. Heterotrophic bacteria also fix N_2_ inside sinking particles in polar regions, albeit at low rates (<1 μmol N m^−2^ day^−1^). We estimate a contribution of 15.3 Tg N year^−1^ of fixed N_2_ by particle-associated heterotrophic bacteria to the global ocean with contributions of 13.1, 0.7, and 1.6 Tg N year^−1^ from tropical, subtropical, and temperate regions, respectively (Fig. 7B). Region-specific estimates are provided in fig. S5. N_2_ fixation extends into the aphotic waters in most parts of the global ocean, while extreme deep water N_2_ fixation occurs mostly in the OMZs and in the North Pacific Ocean (Fig. 7C). Therefore, regions of deep water N_2_ fixation coincide with areas of high contribution from the water column. We find that a nonnegligible amount of N_2_ fixation can extend to 2000-m depth.

## DISCUSSION

Our model shows that N_2_ fixation by heterotrophic bacteria may be widespread in the global oceans, from the tropics to the poles, and from the surface to the abyss. The N_2_ fixation occurs in larger particles, in whose centers the microenvironment allows heterotrophic bacteria to fix N_2_. We find that particle-associated heterotrophic N_2_ fixation can occur under a broad range of temperatures, which explains its wide distribution in the ocean and its importance for the global N_2_ fixation.

Our findings indicate that heterotrophic N_2_ fixation inside particles relies on the formation of anoxic microenvironments, which are more likely to develop in large particles. These results are consistent with previous studies suggesting the formation of anoxic aggregates in the 0.05- to 1-cm size range in the OMZs of the ocean (*39*). However, simultaneous ^15^N-dinitrogen and ^13^C-bicarbonate incubations combined with nanoSIMS showed heterotrophic N_2_ fixation in the oxygenated ocean surface in particles with radius smaller than 0.02 cm (*13*). Size-fractionated metagenomic data revealed a high abundance of heterotrophic diazotrophs in the smallest size fraction (0.2 to 5 μm) of particles (*40*). This suggests that mechanisms beyond anoxic microniches may influence N_2_ fixation in small particles. Therefore, despite the contribution of heterotrophic N_2_ fixers to global N_2_ fixation suggested by our model represents to date the best quantification attempt, we may still underestimate the contribution from small particles.

Most well-known cyanobacterial diazotrophs fix N_2_ at higher temperatures than those estimated for N_2_ fixation by particle-associated heterotrophic bacteria with our model. Low O_2_ solubility and high rates of respiration at high temperatures allow nonsymbiotic cyanobacteria to maintain intracellular anoxic conditions and to carry out N_2_ fixation (*41*). Compared to other cyanobacteria, the symbiotic unicellular cyanobacteria UCYN-A can fix N_2_ at much lower temperatures (*42*, *43*), albeit a mechanistic understanding of N_2_ fixation by this group is still lacking [but see (*44*)]. For particle-associated heterotrophic diazotrophs, high temperature results in a mismatch between increased substrate hydrolysis and poor uptake of hydrolyzed materials, thus restricting N_2_ fixation. The dynamics inside particles allow heterotrophic bacteria to perform N_2_ fixation at much lower temperatures than cyanobacteria. The activity of N_2_ fixation at a broad range of temperatures suggested by our model study explains the success of particle-associated heterotrophic bacteria over a wide range of environmental conditions.

Compared to cyanobacterial diazotrophs, particle-associated heterotrophic diazotrophs show a distinct latitudinal distribution in the oceans. While experimentally observed temperature ranges, in situ measurements of N_2_ fixation rates, and microscopy and quantitative PCR–based enumeration of N_2_ fixers suggest that most cyanobacterial diazotrophs prevail in warm, low latitude tropical, and subtropical surface waters (*31*, *32*, *45*), our model shows that heterotrophic bacteria inside sinking particles fix N_2_ in deep waters at low and high latitudes. We find that the gradients of temperature and O_2_ concentration in the water column interact to determine N_2_ fixation inside sinking particles at different latitudes. This implies the presence of a well-defined niche partitioning between cyanobacterial and particle-associated heterotrophic diazotrophs.

Our results show high particle-associated heterotrophic N_2_ fixation activity in OMZs. Our estimated maximum volumetric N_2_ fixation rate in OMZs is not only comparable to the overall range of measured aphotic N_2_ fixation rates (typically <1 μmol N m^−3^ day^−1^) in the global ocean (*6*), it also matches with the range of measured average volumetric aphotic N_2_ fixation rates in OMZs (0.08 to 1.27 μmol N m^−3^ day^−1^) (*46*–*48*). Our calculated depth-integrated rates lie well within the highest measured aphotic N_2_ fixation rate of 501 μmol N m^−2^ day^−1^ from OMZs (*47*). The high rate of N_2_ fixation we estimate in aphotic waters of OMZs supports the previous observation that deep waters in OMZs can contribute up to 87 to 93% of the whole water column N_2_ fixation (*46*, *47*). Therefore, our results are in line with the general understanding that OMZs are characterized by low volumetric N_2_ fixation rates (*46*, *47*, *49*–*52*) and moderate to high depth-integrated N_2_ fixation rates when considering the whole water column (*46*, *47*). The high N_2_ fixation rates we find in deep waters are also consistent with the dominant and diverse communities of heterotrophic diazotrophs observed in OMZs (*49*). We argue, thus, that OM*Z*s are hotspots of aphotic N_2_ fixation by particle-associated heterotrophic diazotrophs.

OMZs are characterized by water layers of low O_2_ concentrations, situated between a few hundred meters to about 1000 m depth (fig. S4A), and high water temperatures at the surface that decrease with increasing depth (fig. S4C). We suggest that low O_2_ concentrations in OMZs favor the formation of anoxic conditions inside particles and thereby alleviate the energetic demands of N_2_ fixation. Such a decrease in the cost of N_2_ fixation in low O_2_ environments was previously observed for *Azotobacter vinelandii* (*5*) and *Crocosphaera Watsonii* (*53*). Overall, the temperature levels in the oxygen minimum layers maintain an appropriate balance between hydrolyzation and resource uptake rates (sensu Fig. 4) and make them suitable for N_2_ fixation activities by heterotrophic bacteria.

The N_2_ fixation rates found in our study (up to ~200 μmol N m^−2^ day^−1^) are comparable with previously estimated cyanobacterial depth-integrated N_2_ fixation rates from the photic zone of the global ocean, typically in the range of 1 to 100 μmol N m^−2^ day^−1^ (*54*). Moreover, they are comparable to N_2_ fixation rates predicted for the mesopelagic (13 to 134 μmol N m^−2^ day^−1^), estimated by considering the lower-end range of aphotic N_2_ fixation rates and the volume of the mesopelagic zone (*55*). Overall, our estimated contribution accounts for ~10% of the global marine N_2_ fixation and corresponds to ~20% of the nitrogen supplied by *Trichodesmium* (*56*). However, according to our model assumption, heterotrophic diazotrophs can access only the labile part of the particles. N_2_ fixation would occur in deeper waters if heterotrophic diazotrophs were allowed to access semilabile materials. This, in turn, could enhance our estimated contribution of N_2_ fixation in deeper waters. Our results are consistent with previous studies suggesting a large contribution by heterotrophic diazotrophs to water column N_2_ fixation (*57*, *58*).

We find a wide distribution of heterotrophic N_2_ fixation in the global ocean. Our predicted distribution is supported by recent studies showing a similar worldwide distribution of *nifH* genes of heterotrophic bacteria in the global ocean (*4*). Although UCYN-A can fix N_2_ even in cold, high-latitude waters (*42*, *43*), other cyanobacterial N_2_ fixers are active only in warm tropical and subtropical waters. Together, these indications suggest that, as we move from tropical to polar regions, the dominance of the diazotrophic community shifts from cyanobacteria to particle-associated heterotrophic bacteria and UCYN-A. We thus dispute the long-standing paradigms that (i) oceanic N_2_ fixation is exclusively restricted to surface waters of the tropical and subtropical oceans (*59*) and (ii) cyanobacteria are the only important diazotrophs (*60*).

Our study also indicates the occurrence of particle-associated heterotrophic N_2_ fixation in polar regions. These results are supported by the presence of *nifH* genes of heterotrophic bacteria in the Arctic Ocean (*61*, *62*). In nature, most marine heterotrophic bacteria have the cold-inducible RNA chaperone (CspA) protein that allows them to thrive in cold environments (*63*). Our model does not take into account physiological adaptation; thus, the estimated small contribution of fixation rates at the poles (as well as in cold high-latitude and deep waters) to the global budget of N_2_ fixation may be an underestimation. We expect that the inclusion of such adaptation mechanisms would enable heterotrophic diazotrophs to perform N_2_ fixation in colder environments by lowering the optimal temperature of N_2_ fixation.

The fixation of N_2_ by heterotrophic bacteria associated with sinking particles affects oceanic nitrogen and carbon cycling differently than cyanobacteria. By transforming N_2_ into the bioavailable forms of nitrogen, which are vital to phytoplankton communities, cyanobacterial diazotrophs support a considerable portion of oceanic primary production in the sunlit waters of tropical and subtropical oceans and stimulate carbon sequestration (*56*). Because particle-associated N_2_ fixation by heterotrophic bacteria occurs mostly below the surface layers, these organisms are expected to have indirect and delayed impacts on the oceanic nitrogen cycle compared to the direct and immediate effects of cyanobacterial diazotrophs. However, by allowing microbial degradation of sinking particles beyond the exhaustion of organic nitrogen, N_2_ fixation by heterotrophic bacteria may reduce the vertical carbon flux and the oceanic carbon sequestration. Therefore, as N_2_ fixation by heterotrophic bacteria stimulates the uptake of atmospheric CO_2_ by providing bioavailable nitrogen to primary producers, it also reduces carbon sequestration by accelerating the degradation of particles. In essence, our results establish the importance of N_2_ fixation by particle-associated heterotrophic bacteria for the global nitrogen budget and lay down the basis for assessing the importance of these organisms for the sequestration of CO_2_ in the ocean.

Global warming may have a limited impact on deep N_2_ fixation because the expected increase in temperature will be less pronounced in the deep ocean than in the surface ocean (*64*), However, the fixation of N_2_ by particle-associated heterotrophic bacteria in deep waters depends on the export of organic carbon from the photic zone. Ocean warming is expected to reduce phytoplankton productivity in the surface waters of the tropical and subtropical oceans via reduced mixing and reduced nutrient availability and enhance phytoplankton productivity at higher latitudes and in coastal areas (*65*, *66*). Therefore, as the oceans warm up, N_2_ fixation by particle-associated heterotrophic bacteria will increase at high latitudes and in coastal areas. Considering the dominant contribution of particle-associated heterotrophic bacteria in OMZs found in our study, the projected expansion of OMZs (*67*) in response to global warming may further increase the contribution of bioavailable nitrogen from particle-associated heterotrophic bacteria, calling for a reassessment of the biogeography of primary production on a global ocean scale.

## MATERIALS AND METHODS

The present model is developed on the basis of a previous model by Chakraborty *et al.* (*68*). While the previous model mainly focused on explaining the mechanisms of N_2_ fixation inside a particle, the present model aims to (i) examine how temperature affects and regulates N_2_ fixation inside particles to determine the distribution of heterotrophic diazotrophs in the global ocean and (ii) the contribution of particle-associated heterotrophic diazotrophs in the global nitrogen budget. In this process, we extend the previous model by (i) incorporating temperature regulation into important cellular processes and the diffusive exchange of gases and materials, (ii) incorporating the reduction of the size of sinking particles due to the hydrolyzation and consumption of organic materials by bacteria, and (iii) extending the analysis to the global ocean. The interactions between a particle, bacterial cells, and the surrounding environment are described below, and the mathematical equations determining the temporal variations of all the variables are provided in Table 1.

**Table 1.** Equations of the particle model. All quantities vary with time *t* at a distance *r* from the center of the particle. The operator in brackets represents diffusion in spherical coordinates. Descriptions, units, and values of all parameters are provided in table S1.

Variables	Equations	
Bacteria (cells liter^−1^)	$\frac{\partial B}{\partial t}=\mu^{*}\left(G,A,X_{O_{2}},X_{{\text{NO}}_{3}}\right)B-m_{B}B$	41(a)
Labile polysaccharides (μg G liter^−1^)	$\frac{\partial C_{L}}{\partial t}=-J_{C}B$	41(b)
Labile polypeptides (μg A liter^−1^)	$\frac{\partial P_{L}}{\partial t}=-J_{P}B$	41(c)
Glucose (μg G liter^−1^)	$\frac{\partial G}{\partial t}=J_{C}B-J_{G}B+D_{M}\left(\frac{\partial^{2}G}{\partial r^{2}}+\frac{2}{r}\frac{\partial G}{\partial r}\right)$	41(d)
Amino acids (μg A liter^−1^)	$\frac{\partial A}{\partial t}=J_{P}B-J_{A}B+D_{M}\left(\frac{\partial^{2}A}{\partial r^{2}}+\frac{2}{r}\frac{\partial A}{\partial r}\right)$	41(e)
Oxygen (μmol O_2_ liter^−1^)	$\frac{\partial X_{O_{2}}}{\partial t}=-F_{O_{2}}B+{\bar{D}}_{O_{2}}\left(\frac{\partial^{2}X_{O_{2}}}{\partial r^{2}}+\frac{2}{r}\frac{\partial X_{O_{2}}}{\partial r}\right)$	41(f)
Nitrate (μmol NO_3_ liter^−1^)	$\frac{\partial X_{{\text{NO}}_{3}}}{\partial t}=-J_{{\text{NO}}_{3}}B+{\bar{D}}_{{\text{NO}}_{3}}\left(\frac{\partial^{2}X_{NO_{3}}}{\partial r^{2}}+\frac{2}{r}\frac{\partial X_{{\text{NO}}_{3}}}{\partial r}\right)$	41(g)

### The cell model

#### Growth rate of a cell

The growth rate of a bacteria cell depends on the acquisition of carbon (C) supplied from the particle and nitrogen (N) obtained from the particle and through N_2_ fixation, as well as on metabolic expenses in terms of C.

#### Uptake of C and N

The cell obtains C from glucose, while amino acids supply both C and N. The total amount of C available for the cell from monomers (glucose and amino acids) is (units of C per day)

$$J_{\text{DOC}}=f_{G,C}J_{G}+f_{A,C}J_{A}$$ (1)

and the amount of N available from monomer is (N per day)

$$J_{\text{DON}}=f_{A,N}J_{A}$$ (2)

where *J*_G_ and *J*_A_ are uptake rates of glucose and amino acids (see Eqs. 27 and 28), $f_{G,C}$ is the fraction of C in glucose, and $f_{A,C}$ and $f_{A,N}$ are fractions of C and N in amino acids.

The rate of obtaining N through N_2_ fixation is

$$J_{N_{2}}\left(\psi \right)=\psi M_{N_{2}}$$ (3)

where the parameter ψ (0 < ψ < 1) determines the rate of N_2_ fixation, which can happen at a maximum rate $M_{N_{2}}$. Since the concentration of dissolved dinitrogen (N_2_) gas in seawater is unlimited (*69*), N_2_ fixation is assumed to be limited only by the maximum N_2_ fixation rate.

Therefore, the total uptake of C and N from different sources becomes

$$J_{C}=J_{\text{DOC}}$$ (4)

$$J_{N}\left(\psi \right)=J_{\text{DON}}+J_{N_{2}}\left(\psi \right)$$ (5)

#### Costs

Respiratory costs of cellular processes including N_2_ fixation and its associated O_2_ removal is calculated in two separate stages depending on the cellular O_2_ concentration:

#### Case 1: O_2_ concentration is sufficient to maintain aerobic respiration

Aerobic respiration can and cannot depend on limiting substrate concentration (*70*). Here, we assume that the basal respiratory cost $R_{B}x_{B}$ is independent of the limiting substrates and proportional to the mass of the cell $x_{B}$ (μg C). To solubilize particles, particle-attached bacteria produce ectoenzymes that cleave bonds to make molecules small enough to be transported across the bacterial cell membrane. Cleavage is represented by a biomass-specific ectoenzyme production cost $R_{E}$ (*71*). The metabolic costs related to the uptake of hydrolysed products and intracellular processing are assumed to be proportional to the uptake ($J_{i}$): $R_{G}J_{G}$ and $R_{A}J_{A}$ where the $R_{i}$s are costs per unit of resource uptake. Similarly, the metabolic cost associated with N_2_ fixation is assumed as proportional to the N_2_ fixation rate: $R_{N_{2}}\rho_{\text{CN},B}J_{N_{2}}$, where $\rho_{\text{CN},B}$ is the bacterial C:N ratio. If we define all the above costs as direct costs, then the total direct respiratory cost becomes

$$R_{D}\left(\psi \right)=R_{B}x_{B}+R_{E}x_{B}+R_{G}J_{G}+R_{A}J_{A}+R_{N_{2}}\rho_{\text{CN},B}J_{N_{2}}\left(\psi \right)$$ (6)

Indirect costs related to N_2_ fixation arise from the removal of O_2_ from the cell and the production/replenishment of nitrogenase as the enzyme is damaged by O_2_. Here, we assume the indirect cost from the removal of O_2_ by increasing respiration (*72*). To calculate this indirect cost, the concentration of O_2_ present in the cell is estimated as follows.

Since the timescale of O_2_ inside a cell is short, we have assumed a pseudo-steady state inside the cell; the O_2_ diffusion rate inside a cell is always balanced by the respiration rate (*5*), which can be expressed as

$$\rho_{\text{CO}}F_{O_{2}}=R_{D}\left(\psi \right)$$ (7)

Here, $\rho_{\text{CO}}$ is the conversion factor of respiratory O_2_ to C equivalents, and $F_{O_{2}}$ is the actual O_2_ diffusion rate into a cell from the particle and can be calculated as

$$F_{O_{2}}=4\pi r_{B}K_{O_{2}}\left(X_{O_{2}}-X_{O_{2},C}\right)$$ (8)

where $r_{B}$ is the cell radius, $X_{O_{2}}$ is the local O_2_ concentration inside the particle, $X_{O_{2},C}$ is the cellular O_2_ concentration, and $K_{O_{2}}$ is the effective diffusion coefficient of O_2_ over cell membrane layers. The effective diffusion coefficient is calculated following Inomura *et al.* (*5*) in terms of diffusion coefficient inside particles (${\bar{D}}_{O_{2}}$), the diffusivity of cell membrane layers relative to water (ε_m_), the radius of cellular cytoplasm (*r*_C_), and the thickness of cell membrane layers (*L*_m_) as

$$K_{O_{2}}={\bar{D}}_{O_{2}}\frac{\varepsilon_{m}\left(r_{C}+L_{m}\right)}{\varepsilon_{m}r_{C}+L_{m}}$$ (9)

The apparent diffusivity inside particles (${\bar{D}}_{O_{2}}$) is considered as a fraction $f_{O_{2}}$ of the diffusion coefficient in seawater ($D_{O_{2}}$)

$${\bar{D}}_{O_{2}}=f_{O_{2}}D_{O_{2}}$$ (10)

Combining Eqs. 7 and 8 gives the cellular O_2_ concentration $X_{O_{2},C}$ as

$$X_{O_{2},C}=\text{max}\left[0,X_{O_{2}}-\frac{R_{D}\left(\psi \right)}{4\pi r_{B}K_{O_{2}}\rho_{\text{CO}}}\right]$$ (11)

If there is excess O_2_ present in the cell after respiration $\left(X_{O_{2},C}>0\right)$, then the indirect cost of removing the excess O_2_ to be able to perform N_2_ fixation can be written as

$$R_{O_{2}}\left(\psi \right)=H\left(\psi \right)\rho_{\text{CO}}4\pi r_{B}K_{O_{2}}X_{O_{2},C}$$ (12)

where H(ψ) is the Heaviside function

$$H\left(\psi \right)=\left\{ \begin{array}{c} 0,\;\text{if}\;\psi =0\\ 1,\;\text{if}\;\psi >0 \end{array}\right.$$ (13)

Therefore, the total cost of aerobic respiration becomes

$$R_{\text{tot},A}\left(\psi \right)=R_{D}\left(\psi \right)+R_{O_{2}}\left(\psi \right)$$ (14)

#### Case 2: Respiration is limited by O_2_ (anaerobic respiration)

When available O_2_ is insufficient to maintain aerobic respiration ($R_{\text{tot}}\left(\psi \right)>\rho_{\text{CO}}F_{O_{2},\text{max}}$), cells use NO_3_^−^ and SO_4_^2−^ for respiration. The potential NO_3_^−^ uptake, $J_{{\text{NO}}_{3},\text{pot}}$, can be written as

$$J_{{\text{NO}}_{3},\text{pot}}=M_{{\text{NO}}_{3}}\frac{A_{{\text{NO}}_{3}}X_{{\text{NO}}_{3}}}{A_{{\text{NO}}_{3}}X_{{\text{NO}}_{3}}+M_{{\text{NO}}_{3}}}$$ (15)

where $M_{{\text{NO}}_{3}}$ and $A_{{\text{NO}}_{3}}$ are maximum uptake rate and affinity for NO_3_^−^ uptake, respectively. However, the actual rate of NO_3_^−^ uptake, $J_{{\text{NO}}_{3}}$, is determined by cellular respiratory demand and can be written as

$$J_{{\text{NO}}_{3}}=\text{min}\left\{J_{{\text{NO}}_{3},\text{pot}},\text{max}\left[0,\frac{R_{\text{tot},A}\left(\psi \right)-\rho_{\text{CO}}F_{O_{2},\text{max}}}{\rho_{C{\text{NO}}_{3}}}\right]\right\}$$ (16)

where $\rho_{C{\text{NO}}_{3}}$ is the conversion factor of respiratory NO_3_^−^ to C equivalents and the maximum O_2_ diffusion rate into a cell $F_{O_{2},\text{max}}$ can be obtained by equating cellular O_2_ concentration $X_{O_{2},C}$ to zero in Eq. 8 as

$$F_{O_{2},\text{max}}=4\pi r_{B}K_{O_{2}}X_{O_{2}}$$ (17)

Furthermore, in the absence of sufficient NO_3_^−^, the cell uses SO_4_^2−^ as an electron acceptor for respiration. Since the average concentration of SO_4_^2−^ in seawater is 29 mM (*25*), SO_4_^2−^ is assumed as a nonlimiting nutrient for cell growth, and the potential rate of uptake of SO_4_^2−^ is mainly determined by the maximum uptake rate as

$$J_{{\text{SO}}_{4},\text{pot}}=M_{{\text{SO}}_{4}}$$ (18)

where $M_{{\text{SO}}_{4}}$ is the maximum SO_4_^2−^ uptake rate. The actual rate of SO_4_^2−^ uptake, $J_{{\text{SO}}_{4}}$, can be written as

$$\begin{array}{c} J_{{\text{SO}}_{4}}=\text{min}\\ \left\{J_{{\text{SO}}_{4},\text{pot}},\text{max}\left[0,\frac{R_{\text{tot},A}\left(\psi \right)-\rho_{\text{CO}}F_{O_{2},\text{max}}-\rho_{\text{CN}O_{3}}F_{{\text{NO}}_{3},\text{pot}}}{\rho_{C{\text{SO}}_{4}}}\right]\right\} \end{array}$$ (19)

where $\rho_{C{\text{SO}}_{4}}$ is the conversion factor of respiratory SO_4_^2−^ to C equivalents.

According to formulations 16 and 19, NO_3_^−^ and SO_4_^2−^ uptake occurs only when the diffusive flux of O_2_ and both O_2_ and NO_3_^−^ are insufficient to maintain respiration, respectively. In addition, the uptake rates of NO_3_^−^ and SO_4_^2−^ are also regulated according to the cells’ requirements.

Uptakes of NO_3_^−^ and SO_4_^2−^ incur extra metabolic costs $R_{{\text{NO}}_{3}}\rho_{C{\text{NO}}_{3}}J_{{\text{NO}}_{3}}$ and $R_{{\text{SO}}_{4}}\rho_{C{\text{SO}}_{4}}J_{{\text{SO}}_{4}}$, where $R_{{\text{NO}}_{3}}$ and $R_{{\text{SO}}_{4}}$ are costs per unit of NO_3_^−^ and SO_4_^2−^ uptake. Considering these costs, the total respiratory cost of a cell can be written as

$$R_{\text{tot}}\left(\psi \right)=R_{\text{tot},A}\left(\psi \right)+R_{{\text{NO}}_{3}}\rho_{C{\text{NO}}_{3}}J_{{\text{NO}}_{3}}+R_{{\text{SO}}_{4}}\rho_{C{\text{SO}}_{4}}J_{{\text{SO}}_{4}}$$ (20)

#### Synthesis and growth rate

The assimilated C and N are combined to synthesize biomass. The synthesis rate is constrained by the limiting resource (Liebig’s law of the minimum) and by available electron acceptors such that the total flux of C available for growth *J*_tot_ (μg C day^−1^) is

$$\begin{array}{c} J_{\text{tot}}\left(\psi \right)=\text{min}\\ \left[J_{C}-R_{\text{tot}}\left(\psi \right),\;\rho_{\text{CN},B}J_{N}\left(\psi \right),\;\rho_{\text{CO}}F_{O_{2}}+\rho_{C{\text{NO}}_{3}}J_{{\text{NO}}_{3}}+\rho_{C{\text{SO}}_{4}}J_{{\text{SO}}_{4}}\right] \end{array}$$ (21)

Here, the total available C for growth is $J_{C}-R_{\text{tot}}\left(\psi \right)$, the C required to synthesize biomass from N source is $\rho_{\text{CN},B}J_{N}$, and the C equivalent inflow rate of electron acceptors to the cell is $\rho_{\text{CO}}F_{O_{2}}+\rho_{C{\text{NO}}_{3}}J_{{\text{NO}}_{3}}+\rho_{C{\text{SO}}_{4}}J_{{\text{SO}}_{4}}$. We assume that excess C or N is released from the cell instantaneously.

Here, biomass synthesis is not explicitly limited by a maximum synthesis capacity; synthesis is constrained by the C and N uptake in the functional responses (Eqs. 28 and 29). The division rate μ of the cell (day^−1^) can be written as the total flux of C available for growth divided by the C mass of the cell (*x*_B_)

$$\mu \left(\psi \right)=J_{\text{tot}}\left(\psi \right)/x_{B}$$ (22)

The resulting division rate, μ, is a measure of bacterial fitness. We assume that the cell regulates its N_2_ fixation rate depending on the environmental conditions to gain additional N when sufficient organic N is not available from the particle to maximize its growth rate. The optimal value of the parameter regulating N_2_ fixation ψ (0 ≤ ψ ≤ 1) then becomes

$$\psi^{*}=\text{arg}\underset{\psi }{\text{max}}\left\{\mu \left(\psi \right)\right\}$$ (23)

and the corresponding optimal division rate becomes

$$\mu^{*}=\mu \left(\psi^{*}\right)$$ (24)

### The particle model

Next, we allow facultative nitrogen-fixing bacterial cells to grow in a particle of radius *r*_P_ (cm) and volume *V*_P_ (cm^3^). The particle contains bacterial population *B*(*r*) (cells liter^−1^), polysaccharides $C_{P}\left(r\right)$ (μg G liter^−1^), and polypeptides $P_{P}\left(r\right)$ (μg A liter^−1^) at a radial distance *r* (cm) from the center of the particle, where G and A stand for glucose and amino acids. Only fractions *f*_C_ and *f*_P_ of these polymers are assumed as labile [$C_{L}\left(r\right)=f_{C}C_{P}\left(r\right),P_{L}\left(r\right)=f_{P}P_{P}\left(r\right)$], i.e., accessible by bacteria. Bacterial enzymatic hydrolysis converts the labile polysaccharides and polypeptides into monosaccharides (glucose) (*G*; μg G liter^−1^) and amino acids (*A*; μg A liter^−1^) that are efficiently taken up by bacteria. Moreover, the particle contains O_2_, NO_3_^−^, and SO_4_^2−^ with concentrations $X_{O_{2}}\left(r\right)$ (μmol O_2_ liter^−1^), $X_{{\text{NO}}_{3}}\left(r\right)$ (μmol NO_3_ liter^−1^), and $X_{{\text{SO}}_{4}}\left(r\right)$ (μmol SO_4_ liter^−1^). Glucose and amino acids can diffuse out of the particle, whereas O_2_ and NO_3_^−^ can diffuse into the particle from the surrounding environment. Here, we assume that diffusivity depends on the temperature of the water column (see Eq. 32) and is independent of the sinking speed of the particles. Because of the high concentration of SO_4_^2−^ in ocean waters, we assume that SO_4_^2−^ is not diffusion limited inside particles, instead its uptake is limited by the maximum uptake capacity due to cellular physical constraints. The interactions between a particle, cells, and the surrounding environment, in terms of dynamic equations, are provided in Table 1.

We assume that labile polysaccharide (*C*_L_) and polypeptide (*P*_L_) are hydrolyzed into glucose and amino acids at rates *J*_C_ and *J*_P_ with the following functional forms

$$J_{C}=h_{C}\frac{A_{C}C_{L}}{h_{C}+A_{C}C_{L}}$$ (25)

$$J_{P}=h_{P}\frac{A_{P}P_{L}}{h_{P}+A_{P}P_{L}}$$ (26)

where *h*_C_ and *h*_P_ are maximum hydrolysis rates of the carbohydrate and peptide pool, and *A*_C_ and *A*_P_ are respective affinities. *J*_G_ and *J*_A_ denote uptake of glucose and amino acids

$$J_{G}=M_{G}\frac{A_{G}G}{A_{G}G+M_{G}}$$ (27)

$$J_{A}=M_{A}\frac{A_{A}A}{A_{A}A+M_{A}}$$ (28)

where *M*_G_ and *M*_A_ are maximum rates of glucose and amino acids uptakes, whereas *A*_G_ and *A*_A_ are corresponding affinities. Hydrolyzed monomers diffuse out of the particle at a rate *D*_M_.

*m*_B_ represents the mortality rate of bacteria. $F_{O_{2}}$ and $J_{{\text{NO}}_{3}}$ represent the diffusive flux of O_2_ and the consumption rate of NO_3_^−^, respectively, through the bacterial cell membrane. ${\bar{D}}_{O_{2}}$ and ${\bar{D}}_{{\text{NO}}_{3}}$ are diffusion coefficients of O_2_ and NO_3_^−^ inside the particle.

At the center of the particle (*r* = 0) the gradient of all quantities vanishes

$${\left.\frac{\partial G}{\partial r}\right|}_{r=0}={\left.\frac{\partial A}{\partial r}\right|}_{r=0}={\left.\frac{\partial X_{O_{2}}}{\partial r}\right|}_{r=0}={\left.\frac{\partial X_{NO_{3}}}{\partial r}\right|}_{r=0}=0$$ (29)

while at the surface of the particle ($r=r_{P}$), concentrations are determined by the surrounding environment

$$\begin{array}{c} {G|}_{r=r_{P}}=G_{\infty },\;{A|}_{r=r_{P}}=A_{\infty },\;\\ {X_{O_{2}}|}_{r=r_{P}}=X_{O_{2},\infty },\;{X_{{\text{NO}}_{3}}|}_{r=r_{P}}=X_{{\text{NO}}_{3},\infty } \end{array}$$ (30)

with *G*_∞_, *A*_∞_, $X_{O_{2},\infty }$, and $X_{{\text{NO}}_{3},\infty }$ as concentrations of glucose, amino acids, O_2_, and NO_3_^−^ in the environment.

### Temperature dependency of model components

To describe the temperature sensitivity of cellular rates (hydrolyzation, resource uptakes, N_2_ fixation rates, and respiration), we use the Q_10_ rule (*73*–*75*), which describes the factorial change in a rate resulting from a 10°C temperature increase. At temperature *T*, the cellular rate *R*_C_ is related to the base rate *R*_C,0_ at the base temperature *T*_0_ according to

$$R_{C}=R_{C,0}{Q_{10}}^{\frac{T-T_{0}}{10}}$$ (31)

The diffusive exchange of materials (NO_3_^−^, glucose, and amino acids), between particles and their surroundings, and O_2_, between cells and their immediate surroundings, depends strongly on water temperature. To account for the temperature dependency of diffusivity, we followed the Walden’s rule (*27*), expressed by

$$D=D_{0}\eta_{0}\frac{T}{\eta T_{0}}$$ (32)

where *D* and η are, respectively, the diffusivity and the viscosity of water (*28*) at the given temperature *T*. *D*_0_ and η_0_ are diffusion coefficient and viscosity at *T*_0_, respectively.

### Particle size spectrum

The size spectrum of all particles $n\left(r_{P}\right)$ represents the number of particles per unit volume of water per size increment. On the surface ocean, the size spectrum is described by a power law distribution (*19*) of the form

$$n\left(r_{P}\right)=n_{0}{\left(d_{P}/d_{\text{ref}}\right)}^{-\xi }$$ (33)

where $d_{P}\;\left(=2r_{P}\right)$ is the particle diameter, *d*_ref_ is the reference diameter (set to 4 μm in this study), *n*_0_ is the density of particles with respect to the reference diameter *d*_ref_, and ξ, the exponent, represents the relative concentration of small to large particles: the steeper (more negative) the exponent, the greater the proportion of smaller particles, whereas the flatter (less negative) the exponent, the greater the proportion of larger particles. We assume that the particle size spectrum follows this distribution in surface waters, and each size class evolves freely away from the power-law distribution while sinking, depending on the concentration gradients of temperature, O_2_, and NO_3_^−^ and bacterial degradation of particles.

### Particle sinking speed

The sinking speed, *w* (m/day), of a particle of radius *r*_P_ can be written as

$$w=c_{w}{\left(d_{P}\right)}^{\eta }$$ (34)

where η is the dimensionless scaling exponent and *c*_w_ is the prefactor coefficient or the sinking speed of a 1-cm particle (*29*).

### Reduction of particle radius

Because of the hydrolyzation of polymers, particles shrink in size while sinking until they run out of all labile materials. To calculate the radius of a particle at each time step, we use the relationship between total carbon content, *C*_tot_, and the radius, *r*_P_, of the particle (*76*) as

$$C_{\text{tot}}=C_{\text{ref}}{\left(r_{P}/\bar{r_{\text{ref}}}\right)}^{\alpha }$$ (35)

where $\bar{r_{\text{ref}}}$ is the value of the radius of a standard reference particle whose mass is *C*_ref_ and the exponent α represents the fractal dimension of the particle.

The total carbon content of the particle can be obtained as

$$C_{\text{tot}}=\int 4\pi r^{2}C\left(r\right)dr$$ (36)

Here, the carbon content C(r) at a radial distance *r* can be calculated as

$$C\left(r\right)=f_{G,C}C_{P}+f_{A,C}P_{P}$$ (37)

where *C*_P_ and *P*_P_ are the amounts of polysaccharides and polypeptides in the particle, and *f*_G,C_ and *f*_A,C_ are the fractions of carbon in glucose and amino acids, respectively.

Since we assume that bacterial cells cannot access the nonlabile part of the particle, the radius of the particle reduces only due to the reduction in the concentration of the labile part of the polymers, while the concentration of the nonlabile part remains constant. Bacteria stop degrading the particle when all the labile material is depleted.

### *Calculation of total* *N*_2_ *fixation rate*

The model represents a population of facultative heterotrophic diazotrophs that grow at a rate similar to other heterotrophic bacteria. The whole community initiates N_2_ fixation when conditions become suitable. Although under natural conditions, the growth rate of N_2_ fixers always remains low and only constitutes a fraction of the bacterial community (*77*). To avoid overestimating diazotroph cell concentrations and, thus, total N_2_ fixation rates, we assume a fraction σ of the total bacterial population, *B*(*r*) at a radial distance *r*, actively fixes N_2_, i.e., $B_{N_{2}}\left(r\right)=\sigma B\left(r\right)$.

The total amount of fixed N_2_ in a particle of radius *r*_P_ (cm) is (μg N particle^−1^)

$$N_{\text{fix},P}=4\pi \int_{r=0}^{r_{P}}r^{2}B_{N_{2}}\left(r\right)J_{N_{2}}\left(r\right)\;dr$$ (38)

where $J_{N_{2}}\left(r\right)$ is the cellular N_2_ fixation rate at a radial distance *r*.

The *N*_2_ fixation rate per unit volume of water, *N*_fix,V_ (μmol N m^−3^ day^−1^), can be calculated as

$$N_{\text{fix},V}=\frac{1}{14}\int_{x=x_{\min}}^{x_{\max}}N_{\text{fix},P}n\left(r_{P}\right)\;dx$$ (39)

where *x*_min_ and *x*_max_ represent the minimum and maximum sizes (radius) of particles, respectively, and *n*(*r*_*P*_) represents the number of particles per unit volume of water per size increment, which is the size spectrum of particles.

The depth-integrated N_2_ fixation rate, *N*_fix,D_ (μmol N m^−2^ day^−1^), can be obtained by

$$N_{\text{fix},D}\left(t\right)=\int_{z=0}^{Z}N_{\text{fix},V}dz$$ (40)

where *Z* is the depth of the water column.

### Initial setup for numerical simulations.

#### General setup

In our simulations, we consider a heterotrophic bacterial population of cell radius 0.29 μm (*68*) (50 fg C cell^−1^) (*78*) living inside particles. The initial concentrations of polysaccharide and polypeptide are 2.6 × 10^8^ μg G liter^−1^ (*79*) and 1.6 × 10^8^ μg A liter^−1^ (*79*), with labilities of 0.238 (*80*) and 0.5 (*80*), respectively. Outside the particle, the glucose, amino acids, O_2_, NO_3_^−^, and SO_4_^2−^ concentrations are kept fixed at 50 μg G liter^−1^, 5 μg A liter^−1^, 50 μmol O_2_ liter^−1^, 15 μmol NO_3_^−^ liter^−1^, and 29 × 10^3^ μmol SO_4_^2−^ liter^−1^, respectively.

While calculating the thermal range of N_2_ fixation, we allow heterotrophic bacteria to grow inside a particle of radius 0.15 cm under fixed environmental conditions (*76*, *81*). Since our system of differential equations is very stiff, we solve it using a very small time step, 10^−6^ days. Each particle sinks through the water column based on a size-dependent settling velocity, which is reduced at every time step as the particle loses carbon due to hydrolysis. To keep the model simple and focus on the processes affecting the N_2_ fixation, we neglect the process of aggregation and disaggregation.

#### Latitudinal and global distribution

Consistent with what is observed in the global ocean, we allow particles ranging from 5 μm to 0.25 cm (radius) to sink through the water columns (*76*, *81*). The whole size range is divided into 21 size classes of particles.

We consider the global distribution of the abundance of different size classes of particles in the surface ocean. The parameters *n*_0_ and *d*_ref_, defining the particle size distribution in our model (Eq. 33), are kept fixed to values that allow us to match the particle size distribution observed in Monterey Bay, CA (*19*). This simulated distribution is then validated against data from the northern part of the South China Sea (*82*) (fig. S6). The parameter ξ, representing the exponent of the particle size spectrum, varies globally in relation to region-specific particle size distributions at the ocean surface (*30*).

Different types of particles with different origins and characterized by different concentrations of polysaccharides and polypeptides can be found in the oceans. Although in our model the global distribution of particle size at the ocean surface reflects some of the above properties, future studies should consider particle type–specific concentrations of polysaccharides and polypeptides, diffusive exchange of gases and other materials, and sinking speed. Moreover, particles undergo processes of aggregation and disaggregation, which can affect particle size distributions with depth. However, the abundance and proportion of large particles do not appear to change markedly in the mesopelagic part of the ocean (*83*), especially in the OMZs (*84*), where (based on our results) more than 80% of heterotrophic N_2_ fixation occurs. We expect that small variations in the density of macroscopic particles will have minimal impacts on our estimates of particle associated global N_2_ fixation by heterotrophic bacteria. Therefore, to keep our focus on N_2_ fixation and to maintain the computational costs within reasonable limits, we assume that the particle size distribution does not vary with depth.

To examine the latitudinal variation in N_2_ fixation by heterotrophic diazotrophs associated with sinking particles, we chose a transect in the North Pacific Ocean along 137.5°W spanning from 0° to 60°N and allow particles to sink through the water column. We force the model using climatological data from the World Ocean Atlas for the vertical distribution of O_2_ (*22*), NO_3_^−^ (*21*), temperature (*23*) (fig. S7), and latitude-specific particle size distributions.

For the global simulation, the model is run at every 5° by 5° grid point using vertical fields of annual mean temperature, O_2_ and NO_3_^−^ concentrations (from the World Ocean Atlas, as mentioned above), and region-specific particle size distributions at the ocean surface as forcing. These annual data were interpolated to fill in missing values. At each of these locations, we restricted our analysis to the maximum depth at which data were available. We assume that N_2_ fixation stops below that depth. Note that, for a large portion of the Southern Ocean, data were available only for the upper 500 m of the water column. We investigate the global distribution of heterotrophic N_2_ fixation by plotting depth-integrated N_2_ fixation rates. In addition, the contribution to the global nitrogen budget is calculated by integrating depth-integrated N_2_ fixation rates over all the grid points.

## Supplementary Materials

### This PDF file includes:

Supplementary Text Figs. S1 to S7 Table S1 References

## References

[1] N. Gruber, J. N. Galloway, An Earth-system perspective of the global nitrogen cycle. Nature 451, 293–296 (2008). 18202647 10.1038/nature06592

[2] H. Farnelid, A. F. Andersson, S. Bertilsson, W. A. Al-Soud, L. H. Hansen, S. Sørensen, G. F. Steward, Å. Hagström, L. Riemann, Nitrogenase gene amplicons from global marine surface waters are dominated by genes of non-cyanobacteria. PLOS ONE 6, e19223 (2011). 21559425 10.1371/journal.pone.0019223 PMC3084785

[3] D. Bombar, R. W. Paerl, L. Riemann, Marine non-cyanobacterial diazotrophs: Moving beyond molecular detection. Trends Microbiol. 24, 916–927 (2016). 27476748 10.1016/j.tim.2016.07.002

[4] K. A. Turk-Kubo, M. R. Gradoville, S. Cheung, F. M. Cornejo-Castillo, K. J. Harding, M. Morando, M. Mills, J. P. Zehr, Non-cyanobacterial diazotrophs: Global diversity, distribution, ecophysiology, and activity in marine waters. FEMS Microbiol. Rev. 47, fuac046 (2022). 10.1093/femsre/fuac046 PMC10719068 36416813

[5] K. Inomura, J. Bragg, M. J. Follows, A quantitative analysis of the direct and indirect costs of nitrogen fixation: A model based on *Azotobacter vinelandii*. ISME J. 11, 166–175 (2017). 27740611 10.1038/ismej.2016.97 PMC5315487

[6] P. H. Moisander, M. Benavides, S. Bonnet, I. Berman-Frank, A. E. White, L. Riemann, Chasing after non-cyanobacterial nitrogen fixation in marine pelagic environments. Front. Microbiol. 8, 1736 (2017). 28943875 10.3389/fmicb.2017.01736 PMC5596534

[7] H. W. Paerl, L. E. Prufert, Oxygen-poor microzones as potential sites of microbial N2 fixation in nitrogen-depleted aerobic marine waters. Appl. Environ. Microbiol. 53, 1078–1087 (1987). 16347337 10.1128/aem.53.5.1078-1087.1987 PMC203813

[8] S. Hallstrøm, J. B. Raina, M. Ostrowski, D. H. Parks, G. W. Tyson, P. Hugenholtz, R. Stocker, J. R. Seymour, L. Riemann, Chemotaxis may assist marine heterotrophic bacterial diazotrophs to find microzones suitable for N_2_ fixation in the pelagic ocean. ISME J. 16, 2525–2534 (2022). 35915168 10.1038/s41396-022-01299-4 PMC9561647

[9] J. N. Pedersen, D. Bombar, R. W. Paerl, L. Riemann, Diazotrophs and N_2_-Fixation associated with particles in coastal estuarine waters. Front. Microbiol. 9, 2759 (2018). 30505296 10.3389/fmicb.2018.02759 PMC6250843

[10] R. W. Paerl, T. N. G. Hansen, N. N. S. E. Henriksen, A. K. Olesen, L. Riemann, N-fixation and related O2 constraints on model marine diazotroph Pseudomonas stutzeri BAL361. Aquat. Microb. Ecol. 81, 125–136 (2018).

[11] H. Farnelid, K. Turk-Kubo, H. Ploug, J. E. Ossolinski, J. R. Collins, B. A. S. Van Mooy, J. P. Zehr, Diverse diazotrophs are present on sinking particles in the North Pacific Subtropical Gyre. ISME J. 13, 170–182 (2019). 30116043 10.1038/s41396-018-0259-x PMC6299005

[12] E. Geisler, A. Bogler, E. Rahav, E. Bar-Zeev, Direct detection of heterotrophic diazotrophs associated with planktonic aggregates. Sci. Rep. 9, 9288 (2019). 31243322 10.1038/s41598-019-45505-4 PMC6594930

[13] K. J. Harding, K. A. Turk-Kubo, E. W. K. Mak, P. K. Weber, X. Mayali, J. P. Zehr, Cell-specific measurements show nitrogen fixation by particle-attached putative non-cyanobacterial diazotrophs in the North Pacific Subtropical Gyre. Nat. Commun. 13, 6979 (2022). 36379938 10.1038/s41467-022-34585-y PMC9666432

[14] M. Bentzon-Tilia, S. J. Traving, M. Mantikci, H. Knudsen-Leerbeck, J. L. S. Hansen, S. Markager, L. Riemann, Significant N_2_ fixation by heterotrophs, photoheterotrophs and heterocystous cyanobacteria in two temperate estuaries. ISME J. 9, 273–285 (2015). 25026373 10.1038/ismej.2014.119 PMC4303622

[15] C. Martínez-Pérez, W. Mohr, A. Schwedt, J. Dürschlag, C. M. Callbeck, H. Schunck, J. Dekaezemacker, C. R. T. Buckner, G. Lavik, B. M. Fuchs, M. M. M. Kuypers, Metabolic versatility of a novel N2-fixing Alphaproteobacterium isolated from a marine oxygen minimum zone. Environ. Microbiol. 20, 755–768 (2018). 29194930 10.1111/1462-2920.14008

[16] S. A. Rose, B. M. Robicheau, J. Tolman, D. Fonseca-Batista, E. Rowland, D. Desai, J. M. Ratten, E. J. H. Kantor, A. M. Comeau, M. G. I. Langille, J. Jerlström-Hultqvist, E. Devred, G. Sarthou, E. M. Bertrand, J. LaRoche, Nitrogen fixation in the widely distributed marine γ-proteobacterial diazotroph *Candidatus* Thalassolituus haligoni. Sci. Adv. 10, eadn1476 (2024). 39083619 10.1126/sciadv.adn1476 PMC11290528

[17] E. Geisler, A. Bogler, E. Bar-Zeev, E. Rahav, Heterotrophic nitrogen fixation at the hyper-eutrophic Qishon River and estuary system. Front. Microbiol. 11, 2012–2021 (2020). 32670236 10.3389/fmicb.2020.01370 PMC7326945

[18] A. M. P. McDonnell, K. O. Buesseler, Variability in the average sinking velocity of marine particles. Limnol. Oceanogr. 55, 2085–2096 (2010).

[19] G. A. Jackson, R. Maffione, D. K. Costello, A. L. Alldredge, B. E. Logan, H. G. Dam, Particle size spectra between 1 μm and 1 cm at Monterey Bay determined using multiple instruments. Deep. Res. Part I Oceanogr. Res. Pap. 44, 1739–1767 (1997).

[20] D. J. Clements, S. Yang, T. Weber, A. M. P. McDonnell, R. Kiko, L. Stemmann, D. Bianchi, Constraining the particle size distribution of large marine particles in the global ocean with in situ optical observations and supervised learning. Global Biogeochem. Cycles 36, e2021GB007276 (2022).

[21] H. E. Garcia, K. Weathers, C. R. Paver, I. Smolyar, T. P. Boyer, R. A. Locarnini, M. M. Zweng, A. V Mishonov, O. K. Baranova, D. Seidov, J. R. Reagan, World Ocean Atlas 2018, Volume 4: Dissolved Inorganic Nutrients (phosphate, nitrate and nitrate+nitrite, silicate) (2018); https://archimer.ifremer.fr/doc/00651/76336/.

[22] H. E. Garcia, K. Weathers, C. R. Paver, I. Smolyar, T. P. Boyer, R. A. Locarnini, M. M. Zweng, A. V Mishonov, O. K. Baranova, D. Seidov, J. R. Reagan, World Ocean Atlas 2018, Volume 3: Dissolved Oxygen, Apparent Oxygen Utilization, and Oxygen Saturation (2018); https://ncei.noaa.gov/sites/default/files/2020-04/woa18_vol3.pdf.

[23] M. Locarnini, A. Mishonov, O. Baranova, T. Boyer, M. Zweng, H. Garcia, J. Reagan, D. Seidov, K. Weathers, C. Paver, I. Smolyar, World Ocean Atlas 2018, Volume 1: Temperature. *NOAA Atlas NESDIS* 81, 52 (2018).

[24] J. Oelze, Respiratory protection of nitrogenase in Azotobacter species: Is a widely held hypothesis unequivocally supported by experimental evidence? FEMS Microbiol. Rev. 24, 321–333 (2000). 10978541 10.1111/j.1574-6976.2000.tb00545.x

[25] F. J. Millero, *Chemical Oceanography* (CRC Press, ed. 3rd, 2005).

[26] M. A. Maun, *The Biology of Coastal Sand Dunes* (Oxford Univ. Press, 2009).

[27] A. C. Fernandez, G. D. J. Phillies, Temperature dependence of the diffusion coefficient of polystyrene latex spheres. Biopolymers 22, 593–595 (1983).

[28] P. A. Jumars, J. W. Deming, P. S. Hill, L. Karp-Boss, P. L. Yager, W. B. Dade, Physical constraints on marine osmotrophy in an optimal foraging context. Mar.Microb.Food Webs 7, 121–159 (1993).

[29] B. B. Cael, E. L. Cavan, G. L. Britten, Reconciling the size-dependence of marine particle sinking speed. Geophys. Res. Lett. 48, e2020GL091771 (2021).

[30] J. Maerz, K. D. Six, I. Stemmler, S. Ahmerkamp, T. Ilyina, Microstructure and composition of marine aggregates as co-determinants for vertical particulate organic carbon transfer in the global ocean. Biogeosciences 17, 1765–1803 (2020).

[31] F. X. Fu, E. Yu, N. S. Garcia, J. Gale, Y. Luo, E. A. Webb, D. A. Hutchins, Differing responses of marine N2 fixers to warming and consequences for future diazotroph community structure. Aquat. Microb. Ecol. 72, 33–46 (2014).

[32] V. S. Brauer, M. Stomp, C. Rosso, S. A. M. Van Beusekom, B. Emmerich, L. J. Stal, J. Huisman, Low temperature delays timing and enhances the cost of nitrogen fixation in the unicellular cyanobacterium *Cyanothece*. ISME J. 7, 2105–2115 (2013). 23823493 10.1038/ismej.2013.103 PMC3806257

[33] E. Breitbarth, A. Oschlies, J. LaRoche, Physiological constraints on the global distribution of *Trichodesmium* - Effect of temperature on diazotrophy. Biogeosciences 4, 53–61 (2007).

[34] N. Yang, C. A. Merkel, Y.-A. Lin, N. M. Levine, N. J. Hawco, H.-B. Jiang, P.-P. Qu, M. A. DeMers, E. A. Webb, F.-X. Fu, D. A. Hutchins, Warming iron-limited oceans enhance nitrogen fixation and drive biogeographic specialization of the globally important cyanobacterium *Crocosphaera*. Front. Mar. Sci. 8, 628363 (2021).

[35] L. J. Stal, The effect of oxygen concentration and temperature on nitrogenase activity in the heterocystous cyanobacterium *Fischerella* sp. Sci. Rep. 7, 5402 (2017). 28710405 10.1038/s41598-017-05715-0 PMC5511277

[36] V. Fernández-Juárez, E. H. Zech, E. Pol-Pol, N. S. R. Agawin, Cell plasticity of marine mediterranean diazotrophs to climate change factors and nutrient regimes. Diversity 15, 316 (2023).

[37] H. Ploug, M. Kühl, B. Buchholz-Cleven, B. B. Jørgensen, Anoxic aggregates - An ephemeral phenomenon in the pelagic environment? Aquat. Microb. Ecol. 13, 285–294 (1997).

[38] W.-L. Wang, J. K. Moore, A. C. Martiny, F. W. Primeau, Convergent estimates of marine nitrogen fixation. Nature 566, 205–211 (2019). 30760914 10.1038/s41586-019-0911-2

[39] H. Ploug, Small-scale oxygen fluxes and remineralization in sinking aggregates. Limnol. Oceanogr. 46, 1624–1631 (2001).

[40] T. O. Delmont, J. J. Pierella Karlusich, I. Veseli, J. Fuessel, A. M. Eren, R. A. Foster, C. Bowler, P. Wincker, E. Pelletier, Heterotrophic bacterial diazotrophs are more abundant than their cyanobacterial counterparts in metagenomes covering most of the sunlit ocean. ISME J. 16, 927–936 (2022). 34697433 10.1038/s41396-021-01135-1 PMC8941151

[41] L. J. Stal, Is the distribution of nitrogen-fixing cyanobacteria in the oceans related to temperature? Environ. Microbiol. 11, 1632–1645 (2009). 19397684 10.1111/j.1758-2229.2009.00016.x

[42] K. Harding, K. A. Turk-Kubo, R. E. Sipler, M. M. Mills, D. A. Bronk, J. P. Zehr, Symbiotic unicellular cyanobacteria fix nitrogen in the Arctic Ocean. Proc. Natl. Acad. Sci. U.S.A. 115, 13371–13375 (2018). 30538206 10.1073/pnas.1813658115 PMC6310837

[43] T. Shiozaki, A. Fujiwara, K. Inomura, Y. Hirose, F. Hashihama, N. Harada, Biological nitrogen fixation detected under Antarctic sea ice. Nat. Geosci. 13, 729–732 (2020).

[44] T. H. Coale, V. Loconte, K. A. Turk-kubo, B. Vanslembrouck, Y. Takano, T. Nishimura, M. Adachi, M. Le Gros, C. Larabell, J. P. Zehr, Nitrogen-fixing organelle in a marine alga. Science 384, 217–222 (2024). 38603509 10.1126/science.adk1075

[45] Z. Shao, Y. Xu, H. Wang, W. Luo, L. Wang, Y. Huang, N. S. R. Agawin, A. Ahmed, M. Benavides, M. Bentzon-Tilia, I. Berman-Frank, H. Berthelot, I. C. Biegala, M. B. Bif, A. Bode, S. Bonnet, D. A. Bronk, M. V. Brown, L. Campbell, D. G. Capone, E. J. Carpenter, N. Cassar, B. X. Chang, D. Chappell, Y.-l. Lee Chen, M. J. Church, F. M. Cornejo-Castillo, A. M. S. Detoni, S. C. Doney, C. Dupouy, M. Estrada, C. Fernandez, B. Fernández-Castro, D. Fonseca-Batista, R. A. Foster, K. Furuya, N. Garcia, K. Goto, J. Gago, M. R. Gradoville, M. R. Hamersley, B. A. Henke, C. Hörstmann, A. Jayakumar, Z. Jiang, S.-J. Kao, D. M. Karl, L. R. Kittu, A. N. Knapp, S. Kumar, J. L. Roche, H. Liu, J. Liu, C. Lory, C. R. Löscher, E. Marañón, L. F. Messer, M. M. Mills, W. Mohr, P. H. Moisander, C. Mahaffey, R. Moore, B. Mouriño-Carballido, M. R. Mulholland, S.-i. Nakaoka, J. A. Needoba, E. J. Raes, E. Rahav, T. Ramírez-Cárdenas, C. F. Reeder, L. Riemann, V. Riou, J. C. Robidart, V. V. S. S. Sarma, T. Sato, H. Saxena, C. Selden, J. R. Seymour, D. Shi, T. Shiozaki, A. Singh, R. E. Sipler, J. Sun, K. Suzuki, K. Takahashi, Y. Tan, W. Tang, J.-É. Tremblay, K. Turk-Kubo, Z. Wen, A. E. White, S. T. Wilson, T. Yoshida, J. P. Zehr, R. Zhang, Y. Zhang, Y.-W. Luo, Global oceanic diazotroph database version 2 and elevated estimate of global oceanic N2 fixation. Earth Syst. Sci. Data 15, 3673–3709 (2023).

[46] H. Saxena, D. Sahoo, S. Nazirahmed, D. Chaudhari, P. Rahi, S. Kumar, M. Benavides, A. V. Krishna, A. K. Sudheer, A. Singh, The bay of bengal: An enigmatic diazotrophic niche. J. Geophys. Res. Biogeosciences 128, e2023JG007687 (2023).

[47] S. Bonnet, J. Dekaezemacker, K. A. Turk-Kubo, T. Moutin, R. M. Hamersley, O. Grosso, J. P. Zehr, D. G. Capone, Aphotic N2 fixation in the eastern tropical South Pacific Ocean. PLOS ONE 8, e81265 (2013). 24349048 10.1371/journal.pone.0081265 PMC3861260

[48] C. Fernandez, L. Farías, O. Ulloa, Nitrogen fixation in denitrified marine waters. PLOS ONE 6, e20539 (2011). 21687726 10.1371/journal.pone.0020539 PMC3110191

[49] A. Jayakumar, B. X. Chang, B. Widner, P. Bernhardt, M. R. Mulholland, B. B. Ward, Biological nitrogen fixation in the oxygen-minimum region of the eastern tropical North Pacific ocean. ISME J. 11, 2356–2367 (2017). 28742073 10.1038/ismej.2017.97 PMC5607377

[50] A. N. Knapp, K. L. Casciotti, W. M. Berelson, M. G. Prokopenko, D. G. Capone, Low rates of nitrogen fixation in Eastern Tropical South Pacific surface waters. Proc. Natl. Acad. Sci. U.S.A. 113, 4398–4403 (2016). 26976587 10.1073/pnas.1515641113 PMC4843426

[51] C. R. Löscher, W. Mohr, H. W. Bange, D. E. Canfield, No nitrogen fixation in the Bay of Bengal? Biogeosciences 17, 851–864 (2020).

[52] C. F. Reeder, D. L. Arévalo-Martínez, J. A. Carreres-Calabuig, T. Sanders, N. R. Posth, C. R. Löscher, High diazotrophic diversity but low N2 fixation activity in the Northern Benguela upwelling system confirming the enigma of nitrogen fixation in oxygen minimum zone waters. Front. Mar. Sci. 9, 868261 (2022).

[53] T. Großkopf, J. LaRoche, Direct and indirect costs of dinitrogen fixation in Crocosphaera watsonii WH8501 and possible implications for the nitrogen cycle. Front. Microbiol. 3, 236 (2012). 22833737 10.3389/fmicb.2012.00236 PMC3401090

[54] Y. W. Luo, S. C. Doney, L. A. Anderson, M. Benavides, I. Berman-Frank, A. Bode, S. Bonnet, K. H. Boström, D. Böttjer, D. G. Capone, E. J. Carpenter, Y. L. Chen, M. J. Church, J. E. Dore, L. I. Falcón, A. Fernández, R. A. Foster, K. Furuya, F. Gómez, K. Gundersen, A. M. Hynes, D. M. Karl, S. Kitajima, R. J. Langlois, J. Laroche, R. M. Letelier, E. Maranõn, D. J. McGillicuddy, P. H. Moisander, C. M. Moore, B. Mourinõ-Carballido, M. R. Mulholland, J. A. Needoba, K. M. Orcutt, A. J. Poulton, E. Rahav, P. Raimbault, A. P. Rees, L. Riemann, T. Shiozaki, A. Subramaniam, T. Tyrrell, K. A. Turk-Kubo, M. Varela, T. A. Villareal, E. A. Webb, A. E. White, J. Wu, J. P. Zehr, Database of diazotrophs in global ocean: Abundance, biomass and nitrogen fixation rates. Earth Syst. Sci. Data 4, 47–73 (2012).

[55] M. Benavides, S. Bonnet, I. Berman-Frank, L. Riemann, Deep into oceanic N2 fixation. Front. Mar. Sci. 5, 108 (2018).

[56] D. G. Capone, J. P. Zehr, H. W. Paerl, B. Bergman, E. J. Carpenter, Trichodesmium, a globally significant marine cyanobacterium. Science 276, 1221–1229 (1997).

[57] M. Benavides, K. M. Shoemaker, P. H. Moisander, J. Niggemann, T. Dittmar, S. Duhamel, O. Grosso, M. Pujo-Pay, S. Hélias-Nunige, A. Fumenia, S. Bonnet, Aphotic N_2_ fixation along an oligotrophic to ultraoligotrophic transect in the western tropical South Pacific Ocean. Biogeosciences 15, 3107–3119 (2018).

[58] E. Rahav, E. Bar-Zeev, S. Ohayon, H. Elifantz, N. Belkin, B. Herut, M. R. Mulholland, I. Berman-Frank, Dinitrogen fixation in aphotic oxygenated marine environments. Front. Microbiol. 4, 227 (2013). 23986748 10.3389/fmicb.2013.00227 PMC3753716

[59] J. A. Sohm, E. A. Webb, D. G. Capone, Emerging patterns of marine nitrogen fixation. Nat. Rev. Microbiol. 9, 499–508 (2011). 21677685 10.1038/nrmicro2594

[60] J. P. Zehr, Nitrogen fixation by marine cyanobacteria. Trends Microbiol. 19, 162–173 (2011). 21227699 10.1016/j.tim.2010.12.004

[61] L. W. von Friesen, M. L. Paulsen, O. Müller, F. Gründger, L. Riemann, Glacial meltwater and seasonality influence community composition of diazotrophs in Arctic coastal and open waters. FEMS Microbiol. Ecol. 99, fiad067 (2023). 37349965 10.1093/femsec/fiad067

[62] L. W. von Friesen, L. Riemann, Nitrogen fixation in a changing Arctic Ocean: An overlooked source of nitrogen? Front. Microbiol. 11, 596426 (2020). 33391213 10.3389/fmicb.2020.596426 PMC7775723

[63] C. Barria, M. Malecki, C. M. Arraiano, Bacterial adaptation to cold. Microbiology 159, 2437–2443 (2013). 24068238 10.1099/mic.0.052209-0

[64] W. Llovel, J. K. Willis, F. W. Landerer, I. Fukumori, Deep-ocean contribution to sea level and energy budget not detectable over the past decade. Nat. Clim. Chang. 4, 1031–1035 (2014).

[65] G. C. Hays, A. J. Richardson, C. Robinson, Climate change and marine plankton. Trends Ecol. Evol. 20, 337–344 (2005). 16701390 10.1016/j.tree.2005.03.004

[66] P. G. Falkowski, M. J. Oliver, Mix and match: How climate selects phytoplankton. Nat. Rev. Microbiol. 5, 813–819 (2007). 17853908 10.1038/nrmicro1751

[67] Y. Zhou, H. Gong, F. Zhou, Responses of horizontally expanding oceanic oxygen minimum zones to climate change based on observations. Geophys. Res. Lett. 49, e2022GL097724 (2022).

[68] S. Chakraborty, K. H. Andersen, A. W. Visser, K. Inomura, M. J. Follows, L. Riemann, Quantifying nitrogen fixation by heterotrophic bacteria in sinking marine particles. Nat. Commun. 12, 4085 (2021). 34215729 10.1038/s41467-021-23875-6 PMC8253789

[69] M. Maun, *The Biology of Coastal Sand Dunes* (Oxford Univ. Press, Oxford, 2009).

[70] S. J. Pirt, Maintenance energy: A general model for energy-limited and energy-sufficient growth. Arch. Microbiol. 133, 300–302 (1982). 7171288 10.1007/BF00521294

[71] K. A. S. Mislan, C. A. Stock, J. P. Dunne, J. L. Sarmiento, Group behavior among model bacteria influences particulate carbon remineralization depths. J. Mar. Res. 72, 183–218 (2014).

[72] H. Dalton, J. R. Postgate, Effect of oxygen on growth of azotobacter chroococcum in batch and continuous cultures. J. Gen. Microbiol. 54, 463–473 (1968). 5709283 10.1099/00221287-54-3-463

[73] Y. Li, L.-L. Sun, Y.-Y. Sun, Q.-Q. Cha, C.-Y. Li, D.-L. Zhao, X.-Y. Song, M. Wang, A. McMinn, X.-L. Chen, Y.-Z. Zhang, Q.-L. Qin, Extracellular enzyme activity and its implications for organic matter cycling in northern chinese marginal seas. Front. Microbiol. 10, 2137 (2019). 31608022 10.3389/fmicb.2019.02137 PMC6755343

[74] C. Serra-Pompei, G. I. Hagstrom, A. W. Visser, K. H. Andersen, Resource limitation determines temperature response of unicellular plankton communities. Limnol. Oceanogr. 64, 1627–1640 (2019).

[75] R. W. Eppley, Temperature and phytoplankton growth in the sea. Fish. Bull. 70, 1063–1085 (1972).

[76] C. A. Durkin, M. L. Estapa, K. O. Buesseler, Observations of carbon export by small sinking particles in the upper mesopelagic. Mar. Chem. 175, 72–81 (2015).

[77] J. J. P. Karlusich, E. Pelletier, F. Lombard, M. Carsique, E. Dvorak, S. Colin, M. Picheral, F. M. Cornejo-Castillo, S. G. Acinas, R. Pepperkok, E. Karsenti, C. de Vargas, P. Wincker, C. Bowler, R. A. Foster, Global distribution patterns of marine nitrogen-fixers by imaging and molecular methods. Nat. Commun. 12, 4160 (2021). 34230473 10.1038/s41467-021-24299-y PMC8260585

[78] M. Simon, A. L. Alldredge, F. Azam, Bacterial carbon dynamics on marine snow. Mar. Ecol. Prog. Ser. 65, 205–211 (1990).

[79] I. Azúa, M. Unanue, B. Ayo, I. Artolozaga, J. Iriberri, Influence of age of aggregates and prokaryotic abundance on glucose and leucine uptake by heterotrophic marine prokaryotes. Int. Microbiol. 10, 13–18 (2007). 17407056

[80] P. Lopez-Fernandez, S. Bianchelli, A. Pusceddu, A. Calafat, A. Sanchez-Vidal, R. Danovaro, Bioavailability of sinking organic matter in the Blanes canyon and the adjacent open slope (NW Mediterranean Sea). Biogeosciences 10, 3405–3420 (2013).

[81] L. Guidi, G. A. Jackson, L. Stemmann, J. C. Miquel, M. Picheral, G. Gorsky, Relationship between particle size distribution and flux in the mesopelagic zone. Deep. Res. Part I Oceanogr. Res. Pap. 55, 1364–1374 (2008).

[82] Z. Wang, S. Hu, Q. Li, H. Liu, G. Wu, Variability of marine particle size distributions and the correlations with inherent optical properties in the coastal waters of the Northern South China Sea. Remote Sens. 14, 2881 (2022).

[83] T. Devries, J. H. Liang, C. Deutsch, A mechanistic particle flux model applied to the oceanic phosphorus cycle. Biogeosciences 11, 5381–5398 (2014).

[84] R. Kiko, M. Picheral, D. Antoine, M. Babin, L. Berline, T. Biard, E. Boss, P. Brandt, F. Carlotti, S. Christiansen, L. Coppola, L. De Cruz, E. Diamond-riquier, X. D. De Madron, A. Elineau, J. Karstensen, D. Kim, R. M. Lekanoff, F. Lombard, R. M. Lopes, A global marine particle size distribution dataset obtained with the Underwater Vision Profiler 5. Earth Syst. Sci. Data Discuss. 14, 4315–4337 (2022).

[85] D. Bianchi, T. S. Weber, R. Kiko, C. Deutsch, Global niche of marine anaerobic metabolisms expanded by particle microenvironments. Nat. Geosci. 11, 263–268 (2018).

[86] F. A. C. Le Moigne, C. Cisternas-Novoa, J. Piontek, M. Maßmig, A. Engel, On the effect of low oxygen concentrations on bacterial degradation of sinking particles. Sci. Rep. 7, 16722 (2017). 29196721 10.1038/s41598-017-16903-3 PMC5711907

[87] I. Klawonn, S. Bonaglia, V. Brüchert, H. Ploug, Aerobic and anaerobic nitrogen transformation processes in N_2_-fixing cyanobacterial aggregates. ISME J. 9, 1456–1466 (2015). 25575306 10.1038/ismej.2014.232 PMC4438332

[88] D. Boeuf, B. R. Edwards, J. M. Eppley, S. K. Hu, K. E. Poff, A. E. Romano, D. A. Caron, D. M. Karl, E. F. DeLong, Biological composition and microbial dynamics of sinking particulate organic matter at abyssal depths in the oligotrophic open ocean. Proc. Natl. Acad. Sci. U.S.A. 116, 11824–11832 (2019). 31127042 10.1073/pnas.1903080116 PMC6575173

[89] C. L. M. Steenbergen, H. J. Korthals, M. van Nes, Ecological observations on phototrophic sulfur bacteria and the role of these bacteria in the sulfur cycle of monomictic Lake Vechten (The Netherlands). Acta Acad. Abo. 47, 97–115 (1987).

[90] R. F. Vaccaro, S. E. Hicks, H. W. Jannasch, F. G. Carey, The occurrence and role of glucose in seawater. Limnol. Oceanogr. 13, 356–360 (1968).

[91] C. Lee, J. L. Bada, Dissolved amino acids in the equatorial Pacific, the Sargasso Sea, and Biscayne Bay. Limnol. Oceanogr. 22, 502–510 (1977).

[92] J. Wright, A. Colling, “The seawater solution” in *Seawater: Its Composition, Properties and Behaviour* (Elsevier, ed. 2nd, 1995), pp. 85–127.

[93] G. Billen, S. Becquevort, Phytoplankton-bacteria relationship in the Antarctic marine ecosystem. Polar Res. 10, 245–254 (1991).

[94] E. Fouilland, M. Gosselin, R. B. Rivkin, C. Vasseur, B. Mostajir, Nitrogen uptake by heterotrophic bacteria and phytoplankton in Arctic surface waters. J. Plankton Res. 29, 369–376 (2007).

[95] T. Treude, J. Niggemann, J. Kallmeyer, P. Wintersteller, C. J. Schubert, A. Boetius, B. B. Jørgensen, Anaerobic oxidation of methane and sulfate reduction along the Chilean continental margin. Geochim. Cosmochim. Acta 69, 2767–2779 (2005).

[96] R. Kondo, D. B. Nedwell, K. J. Purdy, S. de Queiroz Silva, Detection and enumeration of sulphate-reducing bacteria in estuarine sediments by competitive PCR. Geomicrobiol. J. 21, 145–157 (2004).

[97] M. Bentzon-Tilia, I. Severin, L. H. Hansen, L. Riemann, Genomics and ecophysiology of heterotrophic nitrogen-fixing bacteria isolated from estuarine surface water. MBio 6, e00929 (2015). 26152586 10.1128/mBio.00929-15 PMC4495170

[98] K. J. Flynn, “Incorporating plankton respiration in models of aquatic ecosystem function” in *Respiration in Aquatic Ecosystems*, P. A. del Giorgio, P. J. Williams, Eds. (Oxford Univ. Press, 2005), pp. 248–266.

[99] H. Ploug, H. P. Grossart, F. Azam, B. B. Jørgensen, Photosynthesis, respiration, and carbon turnover in sinking marine snow from surface waters of Southern California Bight: Implications for the carbon cycle in the ocean. Mar. Ecol. Prog. Ser. 179, 1–11 (1999).

[100] A. Paulmier, I. Kriest, A. Oschlies, Stoichiometries of remineralisation and denitrification in global biogeochemical ocean models. Biogeosciences 6, 923–935 (2009).

[101] M. Henze, M. C. M. van Loosdrecht, G. A. Ekama, D. Brdjanovic, *Biological Wastewater Treatment Principles, Modelling and Design* (IWA Publishing, 2008).

[102] H. Ploug, U. Passow, Direct measurement of diffusivity within diatom aggregates containing transparent exopolymer particles. Limnol. Oceanogr. 52, 1–6 (2007).

[103] M. McCabe, T. C. Laurent, Diffusion of oxygen, nitrogen and water in hyaluronate solutions. Biochim. Biophys. Acta 399, 131–138 (1975). 1148273 10.1016/0304-4165(75)90219-6

[104] L. Yuan-Hui, S. Gregory, Diffusion of ions in sea water and in deep-sea sediments. Geochim. Cosmochim. Acta 38, 703–714 (1974).

[105] W. D. Stein, *Channels, Carriers, and Pumps: An Introduction to Membrane Transport* (Academic Press, 1990).

[106] L. Prescott, J. Harley, D. Klein, “Procaryotic cell structure and function” in *Microbiology* (McGrawn Hill, ed. 5th, 2002), pp. 41–73.

[107] S. Lee, J. A. Fuhrman, Relationships between biovolume and biomass of naturally derived marine bacterioplankton. Appl. Environ. Microbiol. 53, 1298–1303 (1987). 16347362 10.1128/aem.53.6.1298-1303.1987 PMC203858

