# Supplementary material for: Particle-associated N2 fixation by heterotrophic bacteria in the global ocean
Source: Sci Adv. 2025 Feb 19;11(8):eadq4693. doi: 10.1126/sciadv.adq4693 (PMC11837998; doi:10.1126/sciadv.adq4693)
Supplement: Supplementary file 1 — Supplementary Text Figs. S1 to S7 Table S1 References [file sciadv.adq4693_sm.pdf]

Science Advances
AAAS

Supplementary Materials for

Particle-associated N_2 fixation by heterotrophic bacteria in the global ocean

Subhendu Chakraborty *et al.*

Corresponding author: Subhendu Chakraborty, subhendu.chakraborty@leibniz-zmt.de

Sci. Adv. **11**, eadq4693 (2025)
DOI: 10.1126/sciadv.adq4693

This PDF file includes:

Supplementary Text
Figs. S1 to S7
Table S1
References

Supplementary Text

Supplementary text 1. Mechanisms of N_2 fixation inside a particle

The overall process of N_2 fixation inside a particle at 17°C is similar to that proposed by Chakraborty et al. (*68*) and briefly summarized below (fig. S1). Bacteria secret ectoenzymes to degrade polysaccharides and polypeptides (fig. S1A,B) into glucose and amino acids. Elevated glucose and amino acids concentrations (fig. S1C,D) increase bacterial abundance (fig. S1E) and community respiration (fig. S1F), and an O_2 free zone develops inside the particle (fig. S1G). The formation of anoxic interiors has been previously observed in both laboratory-made particles (*37*) and particles collected from natural environments (*86*), and also inside suspended cyanobacterial colonies of comparable size (*87*). When amino acids are also exhausted, there are no energetically cheaper means of acquiring nitrogen than N_2 fixation; thus, the presence of sufficient glucose sets the stage for N_2 fixation (fig. S1I) to supplement cells with an alternative source of nitrogen to maintain growth. Under anoxia, bacteria maintain respiration using NO_3^- (fig. S1H) and SO_4^{2-} as electron acceptors. Because of the lower energy yield of NO_3^- and SO_4^{2-} respiration, the bacterial cell division rate remains low during N_2 fixation (fig. S1J). The presence of NO_3^- and SO_4^{2-} reducing bacteria in marine sinking particles is well documented (*88*) and these are generally known to have low growth rates (*89*). When the labile components of polysaccharides and polypeptides are exhausted, glucose limitation terminates N_2 fixation. As a result, cell growth ceases, community respiration drops, and O_2 starts to accumulate in the particle resulting in the disappearance of an anoxic interior. During bacterial degradation, the particle radius is reduced over time until all labile polysaccharides and polypeptides are exhausted and only non-labile materials are left.

Supplementary text 2. Water column NCD N_2 fixation at contrasting latitudes

We applied our model to investigate the vertical distribution of N_2 fixation rates at three latitudes 10° N, 30° N, and 50° N, and along the same longitudinal axis of 140° W (Fig. 7 in the main text). The upper 800 m of these three water columns shows remarkably distinct O_2, NO_3^-, and temperature gradients.

We speculate that, because of the relatively higher temperatures and lower O_2 concentrations in the upper 200 m at 10° N, anoxic interiors are formed inside particles much earlier than at the other locations and, eventually, N_2 fixation starts at lower depths (around 300 m) and reaches very high rates. Because of high temperatures at 30° N, N_2 fixation starts around 400 m but due to the presence of high O_2 concentrations, the cost of O_2 removal is very high, and N_2 fixation is limited. Due to low surface temperatures at 50° N, slow biomass development delays N_2 fixation. The low O_2 environment in this location supports, eventually, a high rate of N_2 fixation.

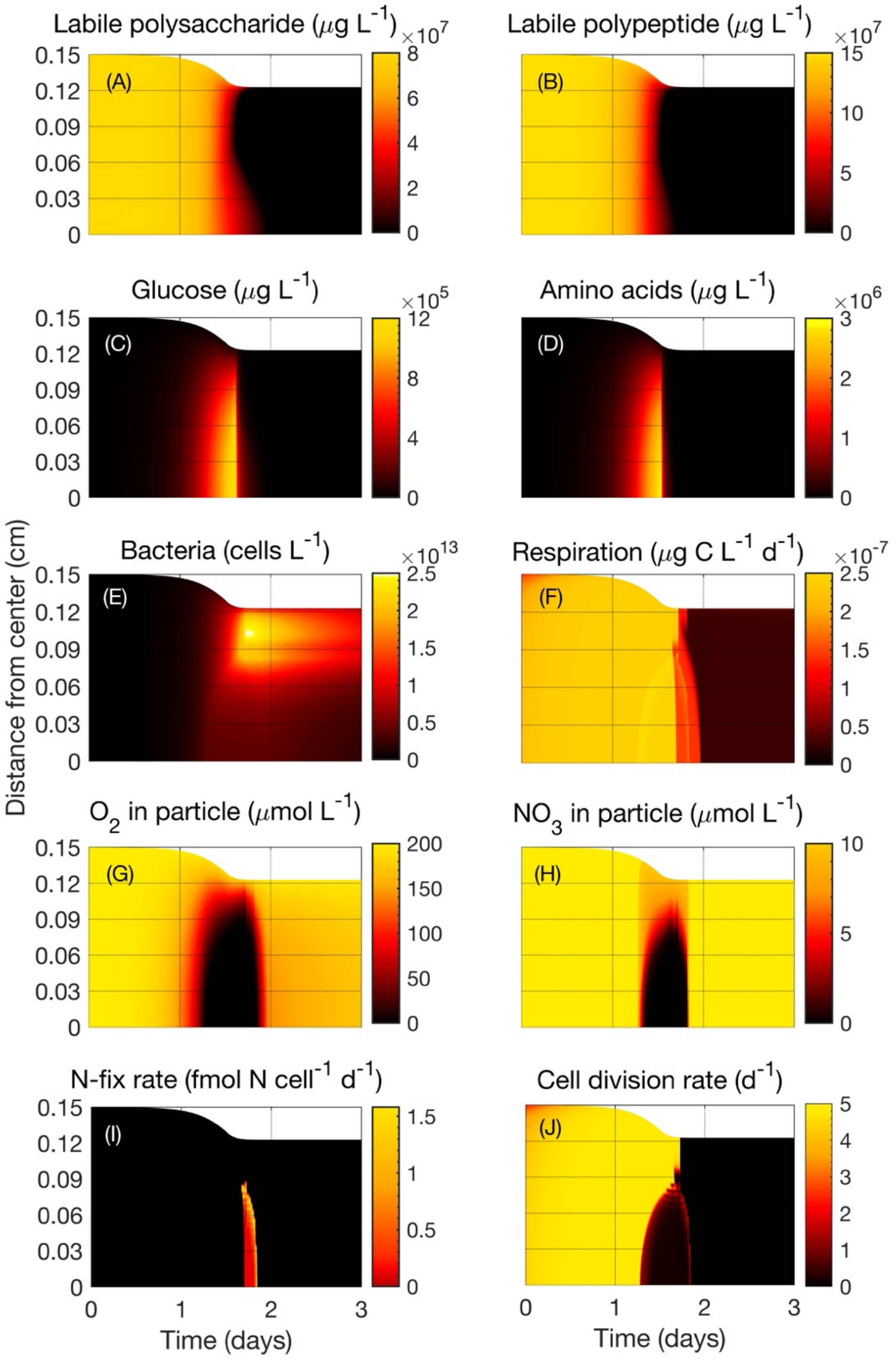

Fig. S1. Temporal dynamics inside a particle of radius 0.15 cm at temperature 17 °C. (A) Labile polysaccharide, (B) labile polypeptide, (C) glucose, (D) amino acids, (E) bacterial abundance, (F) respiration rate, (G) O_2 in particle, (H) NO_3 in particle, (I) N_2 fixation rate, and (J) growth rate as functions of particle radius and time. Because of bacterial degradation, the particle radius decreases with time and stabilizes at a lower value when all labile materials are exhausted. The dynamics are obtained with a surrounding O_2 concentration of 200 μmol O_2 L^{-1} and initial concentrations of labile polysaccharides and polypeptides of, respectively, 8×10^7 μg G L^{-1} and 1.5×10^8 μg A L^{-1}. Other parameters and initial concentrations used to obtain these results are reported in table S1.

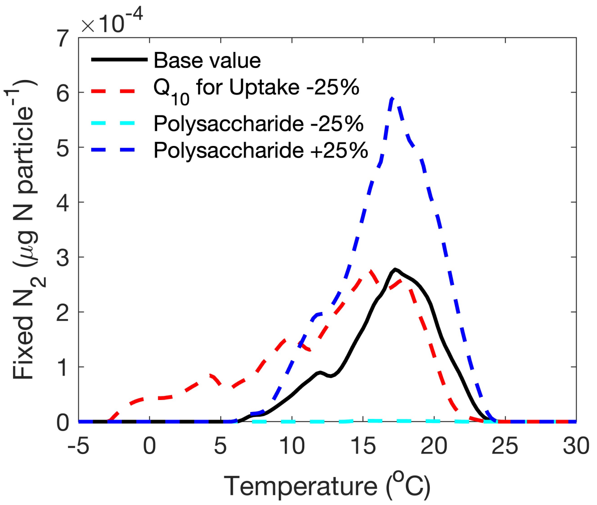

Fig. S2. Sensitivity analysis of the thermal range of N_2 fixation by particle-associated heterotrophic bacteria. The estimated total amount of fixed N_2 at different temperatures in a particle of radius 0.15 cm with a similar setup as in Fig. 2 (black curve), by decreasing the Q_{10} value for bacterial resource uptake (red dashed), by decreasing the initial polysaccharide concentration (cyan dashed), and by increasing the initial polysaccharide concentration (blue dashed) by 25 % from base values.

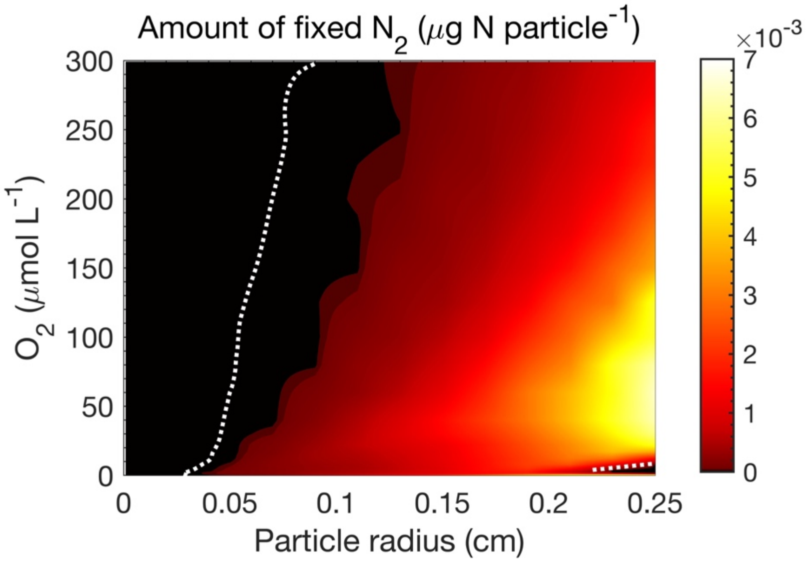

Fig. S3. N_2 fixation for different O_2 concentrations and for different particle sizes. The region within the white dotted lines represents the region of viable N_2 fixation.

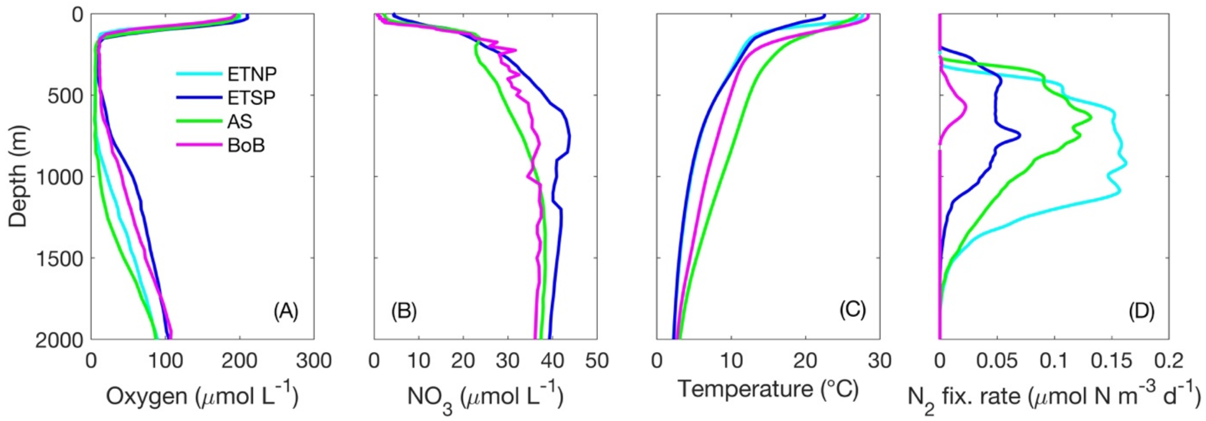

Fig. S4. Environmental factors and N_2 fixation rates through the water column at major oxygen minimum zones of the global ocean. Vertical distributions of (A) O_2, (B) NO_3^{-1}, (C) temperature, and (D) volumetric N_2 fixation rates at the Eastern Tropical North Pacific (ETNP; 12° N, 112° W), at the Eastern Tropical South Pacific (ETSP; 12° S, 87° W), at the Arabian Sea (AS; 17° N, 62° E), and at the Bay of Bengal (BoB; 17° N, 87° E). Vertical gradients of environmental factors are taken from the World Ocean Atlas (*21–23*). Parameters and concentrations used to obtain N_2 fixation rates are reported in table S1.

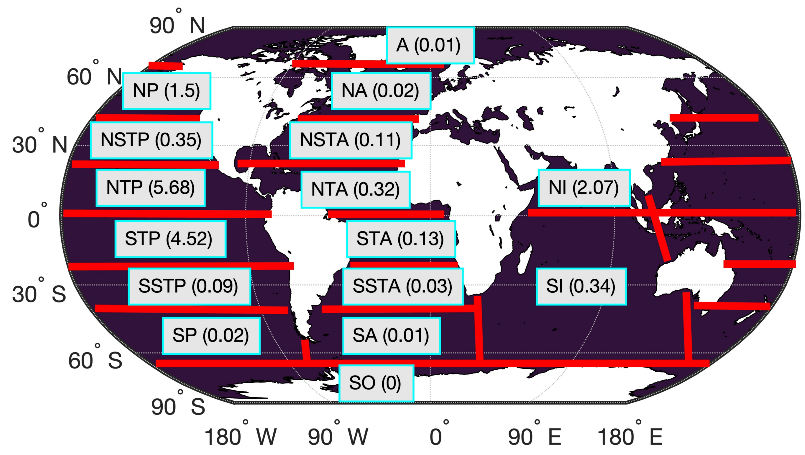

Fig. S5. N_2 fixation rates in different oceanic regions. A (Arctic Ocean), NA (North Atlantic Ocean), NSTA (North Subtropical Atlantic Ocean), NTA (North Tropical Atlantic Ocean), STA (South Tropical Atlantic Ocean), SSTA (South Subtropical Atlantic Ocean), SA (South Atlantic Ocean), SO (Southern Ocean Ocean), NP (North Pacific Ocean), NSTP (North Subtropical Pacific Ocean), NTP (North Tropical Pacific Ocean), STP (South Tropical Pacific Ocean), SSTP (South Subtropical Pacific Ocean), SP (South Pacific Ocean), NI (Northern Indian Ocean), and SI (Southern Indian Ocean). Region specific N_2 fixation rates are reported within brackets in Tg yr^{-1}.

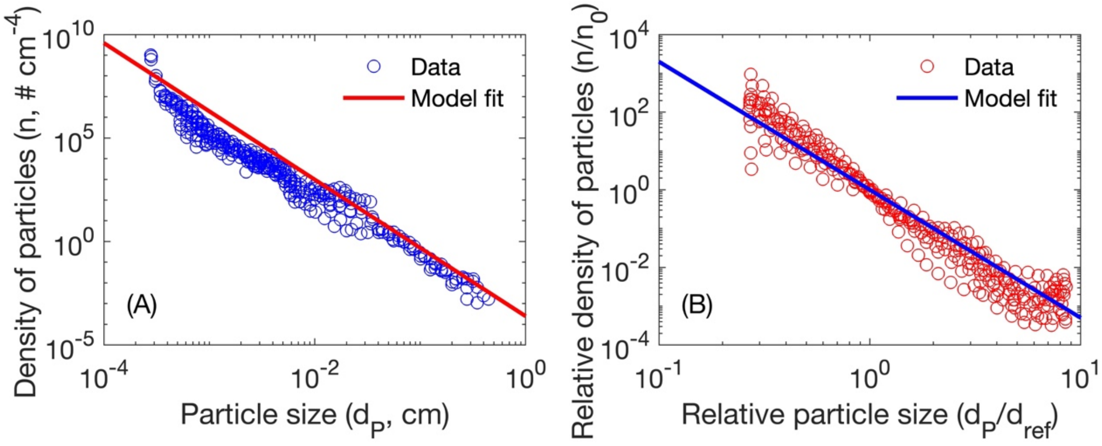

Fig. S6. Particle size distributions. Comparison of simulated particle size distributions (lines) with observed data (circles) at (A) Monterey Bay, CA (*19*), and at (B) northern part of the South China Sea (*82*).

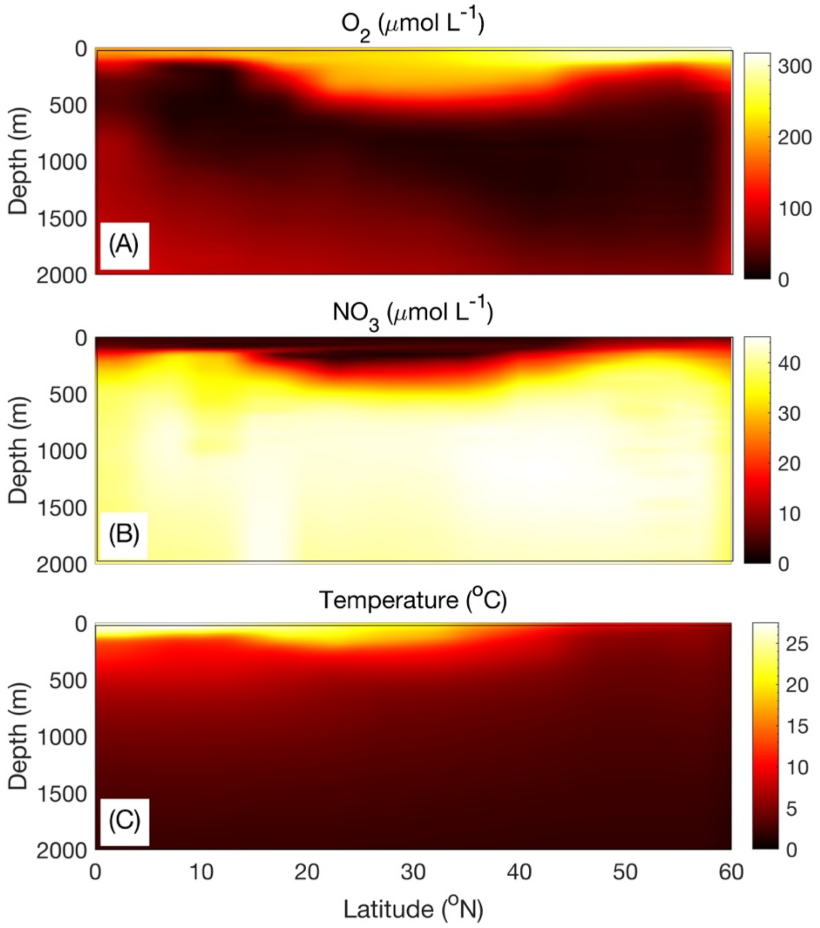

Fig. S7. Vertical distributions of environmental factors along the latitudinal range 0° – 60° N, and at 140° W. Vertical gradients of (A) O_2, (B) NO_3^{-1}, and (C) temperature are taken from World Ocean Atlas (*21–23*).

Table S1. **Initial concentrations of variables and parameter values**. Everything inside the particle is calculated as per liter of particle and outside as per liter of water. G and A indicate glucose and amino acids, respectively.

Symbol	Description	Initial conc./ Parameter value	Unit	Source
C_{P}	Initial polymeric polysaccharide	6.1×10^{7}	µg G L^{-1}	(*79*)
P_{P}	Initial polymeric peptide	7.8×10^{7}	µg A L^{-1}	(*79*)
f_{C}	Labile fraction of C_{P}	0.238	–	(*80*)
f_{P}	Labile fraction of P_{P}	0.5	–	(*80*)
C_{L}	Labile C_{P}	$f_{\mathrm{C}}C_{\mathrm{P}}$	µg G L^{-1}	–
P_{L}	Labile P_{P}	$f_{\mathrm{P}}P_{\mathrm{P}}$	µg A L^{-1}	–
G_{∞}	Glucose conc. outside particle	50	µg L^{-1}	(*90*)
A_{∞}	Amino acids conc. outside particle	5	µg L^{-1}	(*91*)
$X_{\mathrm{O_2},\infty}$	$\mathrm{O_2}$ conc. outside particle	200	µmol O_2 L^{-1}	(*10*)
$X_{\mathrm{NO_3},\infty}$	$\mathrm{NO_3}^-$ conc. outside particle	10	µmol NO_3 L^{-1}	(*85*)
$X_{\mathrm{SO_4},\infty}$	$\mathrm{SO_4}^{2-}$ conc. outside particle	28×10^{3}	µmol SO_4 L^{-1}	(*92*)
G	Initial glucose conc. in particle	G_{∞}	µg L^{-1}	–
A	Initial amino acids conc. in particle	A_{∞}	µg L^{-1}	–
$X_{\mathrm{O_2}}$	Initial $\mathrm{O_2}$ conc. in particle	$X_{\mathrm{O_2},\infty}$	µmol O_2 L^{-1}	–
$X_{\mathrm{O_2},\mathrm{c}}$	Initial $\mathrm{O_2}$ conc. in cell	$X_{\mathrm{O_2},\infty}$	µmol O_2 L^{-1}	–
$X_{\mathrm{NO_3}}$	Initial $\mathrm{NO_3}^-$ conc. in particle	$X_{\mathrm{NO_3},\infty}$	µmol NO_3 L^{-1}	–
$X_{\mathrm{SO_4}}$	Initial $\mathrm{SO_4}^{2-}$ conc. in particle	$X_{\mathrm{SO_4},\infty}$	mmol SO_4 L^{-1}	–
B	Initial bacteria conc. in particle	10^{10}	cells L^{-1}	(*68*)
h_{C}	Max. hydration rate of C_{P}	2.25×10^{-6}	µg cell^{-1} d^{-1}	(*93*)
A_{C}	Affinity of C_{P} hydration	9.0×10^{-9}	L cell^{-1} d^{-1}	(*93*)
h_{P}	Max. hydration rate of P_{P}	1.5×10^{-6}	µg cell^{-1} d^{-1}	(*93*)
A_{P}	Affinity of P_{P} hydration	8.96×10^{-9}	L cell^{-1} d^{-1}	(*93*)
M_{G}	Max. G uptake rate	7.0×10^{-7}	µg cell^{-1} d^{-1}	(*79*)
A_{G}	Affinity for G uptake	2.77×10^{-9}	L cell^{-1} d^{-1}	(*79*)
M_{A}	Max. A uptake rate	4.42×10^{-7}	µg cell^{-1} d^{-1}	(*79*)
A_{A}	Affinity for A uptake	6.95×10^{-9}	L cell^{-1} d^{-1}	(*79*)
$M_{\mathrm{NO_3}}$	Max. $\mathrm{NO_3}^-$ uptake rate	1.63×10^{-9}	µmol NO_3 cell^{-1} d^{-1}	(*94*)
$A_{\mathrm{NO_3}}$	Affinity for $\mathrm{NO_3}^-$ uptake	4.26×10^{-8}	L cell^{-1} d^{-1}	(*95*)
$M_{\mathrm{SO_4}}$	Max. $\mathrm{SO_4}^{2-}$ uptake rate	5×10^{-10}	µmol SO_4 cell^{-1} d^{-1}	(*96*)
$f_{\mathrm{G,C}}$	Fraction of C in G	0.4	–	(*80*)
$f_{\mathrm{A,C}}$	Fraction of C in A	0.445	–	(*91*)
$f_{\mathrm{A,N}}$	Fraction of N in A	0.125	–	(*91*)
$M_{\mathrm{N_2}}$	Max. $\mathrm{N_2}$ fixation rate	5.77×10^{-8}	µg N cell^{-1} d^{-1}	(*97*)
Ψ	Parameter determining $\mathrm{N_2}$ fixation	Variable ($0 < \Psi < 1$)	–	–
R_{B}	Basal maintenance cost	0.05	d^{-1}	(*71*)
R_{E}	Cost of exoenzyme prod.	0.6	d^{-1}	(*71*)
R_{G}	Direct cost of G uptake	0.2	µg C µg C^{-1}	(*98*)
R_{A}	Direct cost of A uptake	0.23	µg C µg C^{-1}	(*98*)
$R_{\mathrm{N_2}}$	Direct cost of $\mathrm{N_2}$ fixation	0.4	µg C µg C^{-1}	(*53*)

$R_{\mathrm{NO_3}}$	Direct cost of NO_3^- uptake	0.4	µg C µg C^{-1}	This study
$R_{\mathrm{SO_4}}$	Direct cost of SO_4^{2-} uptake	0.6	µg C µg C^{-1}	This study
ρ_{CO}	Conversion of respiratory O_2 to C equivalents	10	µg C (µmol O_2)$^{-1}$	(*99*)
$\rho_{\mathrm{CNO_3}}$	Conversion of respiratory NO_3^- to C equivalents	12.5	µg C (µmol NO_3)$^{-1}$	(*100*)
$\rho_{\mathrm{CSO_4}}$	Conversion of respiratory SO_4^{2-} to C equivalents	20	µg C (µmol SO_4)$^{-1}$	(*101*)
$f_{\mathrm{O_2}}$	Fraction of O_2 diffusivity within particles compared to water	0.95	−	(*102*)
$D_{\mathrm{O_2}}$	Diffusion coefficient of O_2 in water	2.12×10^{-5}	cm^2 s^{-1}	(*103*)
$\bar{D}_{\mathrm{O_2}}$	Diffusion coefficient of O_2 inside particles	$f_{\mathrm{O_2}} \times D_{\mathrm{O_2}}$	cm^2 s^{-1}	−
$D_{\mathrm{NO_3}}$	Diffusion coefficient of NO_3^- in water	1.6×10^{-5}	cm^2 s^{-1}	(*104*)
$\bar{D}_{\mathrm{NO_3}}$	Diffusion coefficient of NO_3^- inside particles	$f_{\mathrm{O_2}} \times D_{\mathrm{NO_3}}$	cm^2 s^{-1}	−
D_{M}	Diffusion coeff. of monomers	0.6×10^{-5}	cm^2 s^{-1}	(*105*)
ε_{m}	Diffusivity of cell membrane layers relative to water	7.9×10^{-4}	−	(*5*)
r_{C}	Radius of cellular cytoplasm	0.27	µm	(*68*)
L_{m}	Thickness of cell membrane layer	8×10^{-3}	µm	(*106*)
$\rho_{\mathrm{CN},B}$	Bacterial C:N	3.7	µg C µg N^{-1}	(*107*)
m_B	Mortality rate	0.1	d^{-1}	(*93*)
x_B	Mass of bacteria	5×10^{-8}	µg C	(*78*)
r_B	Radius of bacteria	0.29	µm	(*68*)
V	Volume of bacteria	0.1058	µm^3	(*68*)
r_P	Radius of particle	0.15	cm	(*85*)
σ	Fraction of diazotrophs compared to total bacteria	0.01	−	(*77*)
d_{ref}	Reference particle diameter for particle size spectrum	4	µm	Calibrated
n_0	Particle number at reference diameter	4×10^{-4}	# particle cm^{-4}	Calibrated
ξ	Number spectral slope	3.3	−	(*19*)
w	Particle sinking speed	−	−	−
c_w	Prefactor coefficient	129	m d^{-1} cm$^{-\eta}$	(*29*)
η	Scaling exponent of w	0.63	−	(*29*)
α	Fractal dimension of particle	2.81	−	(*76*)
$\overline{r_{\mathrm{ref}}}$	Radius of reference particle	0.25	cm	This study
C_{ref}	Mass of reference particle	6.25×10^3	µg C	Eq. (7)
T_{ref}	Reference temperature	20	°C	(*97*)
$Q_{10,h}$	Q_{10} value for hydrolysis	2	−	(*73*)
$Q_{10,A}$	Q_{10} value for affinity	1.5	−	(*74*)
$Q_{10,R}$	Q_{10} value for respiration	2	−	(*75*)

REFERENCES AND NOTES

1. N. Gruber, J. N. Galloway, An Earth-system perspective of the global nitrogen cycle. *Nature* **451**, 293–296 (2008).

2. H. Farnelid, A. F. Andersson, S. Bertilsson, W. A. Al-Soud, L. H. Hansen, S. Sørensen, G. F. Steward, Å. Hagström, L. Riemann, Nitrogenase gene amplicons from global marine surface waters are dominated by genes of non-cyanobacteria. *PLOS ONE* **6**, e19223 (2011).

3. D. Bombar, R. W. Paerl, L. Riemann, Marine non-cyanobacterial diazotrophs: Moving beyond molecular detection. *Trends Microbiol.* **24**, 916–927 (2016).

4. K. A. Turk-Kubo, M. R. Gradoville, S. Cheung, F. M. Cornejo-Castillo, K. J. Harding, M. Morando, M. Mills, J. P. Zehr, Non-cyanobacterial diazotrophs: Global diversity, distribution, ecophysiology, and activity in marine waters. *FEMS Microbiol. Rev.* **47**, fuac046 (2022).

5. K. Inomura, J. Bragg, M. J. Follows, A quantitative analysis of the direct and indirect costs of nitrogen fixation: A model based on *Azotobacter vinelandii*. *ISME J.* **11**, 166–175 (2017).

6. P. H. Moisander, M. Benavides, S. Bonnet, I. Berman-Frank, A. E. White, L. Riemann, Chasing after non-cyanobacterial nitrogen fixation in marine pelagic environments. *Front. Microbiol.* **8**, 1736 (2017).

7. H. W. Paerl, L. E. Prufert, Oxygen-poor microzones as potential sites of microbial N2 fixation in nitrogen-depleted aerobic marine waters. *Appl. Environ. Microbiol.* **53**, 1078–1087 (1987).

8. S. Hallstrøm, J. B. Raina, M. Ostrowski, D. H. Parks, G. W. Tyson, P. Hugenholtz, R. Stocker, J. R. Seymour, L. Riemann, Chemotaxis may assist marine heterotrophic bacterial diazotrophs to find microzones suitable for N_2 fixation in the pelagic ocean. *ISME J.* **16**, 2525–2534 (2022).

9. J. N. Pedersen, D. Bombar, R. W. Paerl, L. Riemann, Diazotrophs and N_2-Fixation associated with particles in coastal estuarine waters. *Front. Microbiol.* **9**, 2759 (2018).

10. R. W. Paerl, T. N. G. Hansen, N. N. S. E. Henriksen, A. K. Olesen, L. Riemann, N-fixation and related O2 constraints on model marine diazotroph Pseudomonas stutzeri BAL361. *Aquat. Microb. Ecol.* **81**, 125–136 (2018).

11. H. Farnelid, K. Turk-Kubo, H. Ploug, J. E. Ossolinski, J. R. Collins, B. A. S. Van Mooy, J. P. Zehr, Diverse diazotrophs are present on sinking particles in the North Pacific Subtropical Gyre. *ISME J.* **13**, 170–182 (2019).

12. E. Geisler, A. Bogler, E. Rahav, E. Bar-Zeev, Direct detection of heterotrophic diazotrophs associated with planktonic aggregates. *Sci. Rep.* **9**, 9288 (2019).

13. K. J. Harding, K. A. Turk-Kubo, E. W. K. Mak, P. K. Weber, X. Mayali, J. P. Zehr, Cell-specific measurements show nitrogen fixation by particle-attached putative non-cyanobacterial diazotrophs in the North Pacific Subtropical Gyre. *Nat. Commun.* **13**, 6979 (2022).

14. M. Bentzon-Tilia, S. J. Traving, M. Mantikci, H. Knudsen-Leerbeck, J. L. S. Hansen, S. Markager, L. Riemann, Significant N_2 fixation by heterotrophs, photoheterotrophs and heterocystous cyanobacteria in two temperate estuaries. *ISME J.* **9**, 273–285 (2015).

15. C. Martínez-Pérez, W. Mohr, A. Schwedt, J. Dürschlag, C. M. Callbeck, H. Schunck, J. Dekaezemacker, C. R. T. Buckner, G. Lavik, B. M. Fuchs, M. M. M. Kuypers, Metabolic versatility of a novel N2-fixing Alphaproteobacterium isolated from a marine oxygen minimum zone. *Environ. Microbiol.* **20**, 755–768 (2018).

16. S. A. Rose, B. M. Robicheau, J. Tolman, D. Fonseca-Batista, E. Rowland, D. Desai, J. M. Ratten, E. J. H. Kantor, A. M. Comeau, M. G. I. Langille, J. Jerlström-Hultqvist, E. Devred, G. Sarthou, E. M. Bertrand, J. LaRoche, Nitrogen fixation in the widely distributed marine γ-proteobacterial diazotroph *Candidatus* Thalassolituus haligoni. *Sci. Adv.* **10**, eadn1476 (2024).

17. E. Geisler, A. Bogler, E. Bar-Zeev, E. Rahav, Heterotrophic nitrogen fixation at the hyper-eutrophic Qishon River and estuary system. *Front. Microbiol.* **11**, 2012–2021 (2020).

18. A. M. P. McDonnell, K. O. Buesseler, Variability in the average sinking velocity of marine particles. *Limnol. Oceanogr.* **55**, 2085–2096 (2010).

19. G. A. Jackson, R. Maffione, D. K. Costello, A. L. Alldredge, B. E. Logan, H. G. Dam, Particle size spectra between 1 μm and 1 cm at Monterey Bay determined using multiple instruments. *Deep. Res. Part I Oceanogr. Res. Pap.* **44**, 1739–1767 (1997).

20. D. J. Clements, S. Yang, T. Weber, A. M. P. McDonnell, R. Kiko, L. Stemmann, D. Bianchi, Constraining the particle size distribution of large marine particles in the global ocean with in situ optical observations and supervised learning. *Global Biogeochem. Cycles* **36**, e2021GB007276 (2022).

21. H. E. Garcia, K. Weathers, C. R. Paver, I. Smolyar, T. P. Boyer, R. A. Locarnini, M. M. Zweng, A. V Mishonov, O. K. Baranova, D. Seidov, J. R. Reagan, World Ocean Atlas 2018, Volume 4: Dissolved Inorganic Nutrients (phosphate, nitrate and nitrate+nitrite, silicate) (2018); https://archimer.ifremer.fr/doc/00651/76336/.

22. H. E. Garcia, K. Weathers, C. R. Paver, I. Smolyar, T. P. Boyer, R. A. Locarnini, M. M. Zweng, A. V Mishonov, O. K. Baranova, D. Seidov, J. R. Reagan, World Ocean Atlas 2018, Volume 3: Dissolved Oxygen, Apparent Oxygen Utilization, and Oxygen Saturation (2018); https://ncei.noaa.gov/sites/default/files/2020-04/woa18_vol3.pdf.

23. M. Locarnini, A. Mishonov, O. Baranova, T. Boyer, M. Zweng, H. Garcia, J. Reagan, D. Seidov, K. Weathers, C. Paver, I. Smolyar, World Ocean Atlas 2018, Volume 1: Temperature. *NOAA Atlas NESDIS* 81, 52 (2018).

24. J. Oelze, Respiratory protection of nitrogenase in Azotobacter species: Is a widely held hypothesis unequivocally supported by experimental evidence? *FEMS Microbiol. Rev.* **24**, 321–333 (2000).

25. F. J. Millero, *Chemical Oceanography* (CRC Press, ed. 3rd, 2005).

26. M. A. Maun, *The Biology of Coastal Sand Dunes* (Oxford Univ. Press, 2009).

27. A. C. Fernandez, G. D. J. Phillies, Temperature dependence of the diffusion coefficient of polystyrene latex spheres. *Biopolymers* **22**, 593–595 (1983).

28. P. A. Jumars, J. W. Deming, P. S. Hill, L. Karp-Boss, P. L. Yager, W. B. Dade, Physical constraints on marine osmotrophy in an optimal foraging context. *Mar.Microb.Food Webs* **7**, 121–159 (1993).

29. B. B. Cael, E. L. Cavan, G. L. Britten, Reconciling the size-dependence of marine particle sinking speed. *Geophys. Res. Lett.* **48**, e2020GL091771 (2021).

30. J. Maerz, K. D. Six, I. Stemmler, S. Ahmerkamp, T. Ilyina, Microstructure and composition of marine aggregates as co-determinants for vertical particulate organic carbon transfer in the global ocean. *Biogeosciences* **17**, 1765–1803 (2020).

31. F. X. Fu, E. Yu, N. S. Garcia, J. Gale, Y. Luo, E. A. Webb, D. A. Hutchins, Differing responses of marine N2 fixers to warming and consequences for future diazotroph community structure. *Aquat. Microb. Ecol.* **72**, 33–46 (2014).

32. V. S. Brauer, M. Stomp, C. Rosso, S. A. M. Van Beusekom, B. Emmerich, L. J. Stal, J. Huisman, Low temperature delays timing and enhances the cost of nitrogen fixation in the unicellular cyanobacterium *Cyanothece*. *ISME J.* **7**, 2105–2115 (2013).

33. E. Breitbarth, A. Oschlies, J. LaRoche, Physiological constraints on the global distribution of *Trichodesmium* - Effect of temperature on diazotrophy. *Biogeosciences* **4**, 53–61 (2007).

34. N. Yang, C. A. Merkel, Y.-A. Lin, N. M. Levine, N. J. Hawco, H.-B. Jiang, P.-P. Qu, M. A. DeMers, E. A. Webb, F.-X. Fu, D. A. Hutchins, Warming iron-limited oceans enhance nitrogen fixation and drive biogeographic specialization of the globally important cyanobacterium *Crocosphaera*. *Front. Mar. Sci.* **8**, 628363 (2021).

35. L. J. Stal, The effect of oxygen concentration and temperature on nitrogenase activity in the heterocystous cyanobacterium *Fischerella* sp. *Sci. Rep.* **7**, 5402 (2017).

36. V. Fernández-Juárez, E. H. Zech, E. Pol-Pol, N. S. R. Agawin, Cell plasticity of marine mediterranean diazotrophs to climate change factors and nutrient regimes. *Diversity* **15**, 316 (2023).

37. H. Ploug, M. Kühl, B. Buchholz-Cleven, B. B. Jørgensen, Anoxic aggregates - An ephemeral phenomenon in the pelagic environment? *Aquat. Microb. Ecol.* **13**, 285–294 (1997).

38. W.-L. Wang, J. K. Moore, A. C. Martiny, F. W. Primeau, Convergent estimates of marine nitrogen fixation. *Nature* **566**, 205–211 (2019).

39. H. Ploug, Small-scale oxygen fluxes and remineralization in sinking aggregates. *Limnol. Oceanogr.* **46**, 1624–1631 (2001).

40. T. O. Delmont, J. J. Pierella Karlusich, I. Veseli, J. Fuessel, A. M. Eren, R. A. Foster, C. Bowler, P. Wincker, E. Pelletier, Heterotrophic bacterial diazotrophs are more abundant than their cyanobacterial counterparts in metagenomes covering most of the sunlit ocean. *ISME J.* **16**, 927–936 (2022).

41. L. J. Stal, Is the distribution of nitrogen-fixing cyanobacteria in the oceans related to temperature? *Environ. Microbiol.* **11**, 1632–1645 (2009).

42. K. Harding, K. A. Turk-Kubo, R. E. Sipler, M. M. Mills, D. A. Bronk, J. P. Zehr, Symbiotic unicellular cyanobacteria fix nitrogen in the Arctic Ocean. *Proc. Natl. Acad. Sci. U.S.A.* **115**, 13371–13375 (2018).

43. T. Shiozaki, A. Fujiwara, K. Inomura, Y. Hirose, F. Hashihama, N. Harada, Biological nitrogen fixation detected under Antarctic sea ice. *Nat. Geosci.* **13**, 729–732 (2020).

44. T. H. Coale, V. Loconte, K. A. Turk-kubo, B. Vanslembrouck, Y. Takano, T. Nishimura, M. Adachi, M. Le Gros, C. Larabell, J. P. Zehr, Nitrogen-fixing organelle in a marine alga. *Science* **384**, 217–222 (2024).

45. Z. Shao, Y. Xu, H. Wang, W. Luo, L. Wang, Y. Huang, N. S. R. Agawin, A. Ahmed, M. Benavides, M. Bentzon-Tilia, I. Berman-Frank, H. Berthelot, I. C. Biegala, M. B. Bif, A. Bode, S. Bonnet, D. A. Bronk, M. V. Brown, L. Campbell, D. G. Capone, E. J. Carpenter, N.

Cassar, B. X. Chang, D. Chappell, Y.-l. Lee Chen, M. J. Church, F. M. Cornejo-Castillo, A. M. S. Detoni, S. C. Doney, C. Dupouy, M. Estrada, C. Fernandez, B. Fernández-Castro, D. Fonseca-Batista, R. A. Foster, K. Furuya, N. Garcia, K. Goto, J. Gago, M. R. Gradoville, M. R. Hamersley, B. A. Henke, C. Hörstmann, A. Jayakumar, Z. Jiang, S.-J. Kao, D. M. Karl, L. R. Kittu, A. N. Knapp, S. Kumar, J. L. Roche, H. Liu, J. Liu, C. Lory, C. R. Löscher, E. Marañón, L. F. Messer, M. M. Mills, W. Mohr, P. H. Moisander, C. Mahaffey, R. Moore, B. Mouriño-Carballido, M. R. Mulholland, S.-i. Nakaoka, J. A. Needoba, E. J. Raes, E. Rahav, T. Ramírez-Cárdenas, C. F. Reeder, L. Riemann, V. Riou, J. C. Robidart, V. V. S. S. Sarma, T. Sato, H. Saxena, C. Selden, J. R. Seymour, D. Shi, T. Shiozaki, A. Singh, R. E. Sipler, J. Sun, K. Suzuki, K. Takahashi, Y. Tan, W. Tang, J.-É. Tremblay, K. Turk-Kubo, Z. Wen, A. E. White, S. T. Wilson, T. Yoshida, J. P. Zehr, R. Zhang, Y. Zhang, Y.-W. Luo, Global oceanic diazotroph database version 2 and elevated estimate of global oceanic N2 fixation. *Earth Syst. Sci. Data* **15**, 3673–3709 (2023).

46. H. Saxena, D. Sahoo, S. Nazirahmed, D. Chaudhari, P. Rahi, S. Kumar, M. Benavides, A. V. Krishna, A. K. Sudheer, A. Singh, The bay of bengal: An enigmatic diazotrophic niche. *J. Geophys. Res. Biogeosciences* **128**, e2023JG007687 (2023).

47. S. Bonnet, J. Dekaezemacker, K. A. Turk-Kubo, T. Moutin, R. M. Hamersley, O. Grosso, J. P. Zehr, D. G. Capone, Aphotic N2 fixation in the eastern tropical South Pacific Ocean. *PLOS ONE* **8**, e81265 (2013).

48. C. Fernandez, L. Farías, O. Ulloa, Nitrogen fixation in denitrified marine waters. *PLOS ONE* **6**, e20539 (2011).

49. A. Jayakumar, B. X. Chang, B. Widner, P. Bernhardt, M. R. Mulholland, B. B. Ward, Biological nitrogen fixation in the oxygen-minimum region of the eastern tropical North Pacific ocean. *ISME J.* **11**, 2356–2367 (2017).

50. A. N. Knapp, K. L. Casciotti, W. M. Berelson, M. G. Prokopenko, D. G. Capone, Low rates of nitrogen fixation in Eastern Tropical South Pacific surface waters. *Proc. Natl. Acad. Sci. U.S.A.* **113**, 4398–4403 (2016).

51. C. R. Löscher, W. Mohr, H. W. Bange, D. E. Canfield, No nitrogen fixation in the Bay of Bengal? *Biogeosciences* **17**, 851–864 (2020).

52. C. F. Reeder, D. L. Arévalo-Martínez, J. A. Carreres-Calabuig, T. Sanders, N. R. Posth, C. R. Löscher, High diazotrophic diversity but low N2 fixation activity in the Northern Benguela upwelling system confirming the enigma of nitrogen fixation in oxygen minimum zone waters. *Front. Mar. Sci.* **9**, 868261 (2022).

53. T. Großkopf, J. LaRoche, Direct and indirect costs of dinitrogen fixation in Crocosphaera watsonii WH8501 and possible implications for the nitrogen cycle. *Front. Microbiol.* **3**, 236 (2012).

54. Y. W. Luo, S. C. Doney, L. A. Anderson, M. Benavides, I. Berman-Frank, A. Bode, S. Bonnet, K. H. Boström, D. Böttjer, D. G. Capone, E. J. Carpenter, Y. L. Chen, M. J. Church, J. E. Dore, L. I. Falcón, A. Fernández, R. A. Foster, K. Furuya, F. Gómez, K. Gundersen, A. M. Hynes, D. M. Karl, S. Kitajima, R. J. Langlois, J. Laroche, R. M. Letelier, E. Maranõn, D. J. McGillicuddy, P. H. Moisander, C. M. Moore, B. Mourinõ-Carballido, M. R. Mulholland, J. A. Needoba, K. M. Orcutt, A. J. Poulton, E. Rahav, P. Raimbault, A. P. Rees, L. Riemann, T. Shiozaki, A. Subramaniam, T. Tyrrell, K. A. Turk-Kubo, M. Varela, T. A. Villareal, E. A. Webb, A. E. White, J. Wu, J. P. Zehr, Database of diazotrophs in global ocean: Abundance, biomass and nitrogen fixation rates. *Earth Syst. Sci. Data* **4**, 47–73 (2012).

55. M. Benavides, S. Bonnet, I. Berman-Frank, L. Riemann, Deep into oceanic N2 fixation. *Front. Mar. Sci.* **5**, 108 (2018).

56. D. G. Capone, J. P. Zehr, H. W. Paerl, B. Bergman, E. J. Carpenter, Trichodesmium, a globally significant marine cyanobacterium. *Science* **276**, 1221–1229 (1997).

57. M. Benavides, K. M. Shoemaker, P. H. Moisander, J. Niggemann, T. Dittmar, S. Duhamel, O. Grosso, M. Pujo-Pay, S. Hélias-Nunige, A. Fumenia, S. Bonnet, Aphotic N_2 fixation along an oligotrophic to ultraoligotrophic transect in the western tropical South Pacific Ocean. *Biogeosciences* **15**, 3107–3119 (2018).

58. E. Rahav, E. Bar-Zeev, S. Ohayon, H. Elifantz, N. Belkin, B. Herut, M. R. Mulholland, I. Berman-Frank, Dinitrogen fixation in aphotic oxygenated marine environments. *Front. Microbiol.* **4**, 227 (2013).

59. J. A. Sohm, E. A. Webb, D. G. Capone, Emerging patterns of marine nitrogen fixation. *Nat. Rev. Microbiol.* **9**, 499–508 (2011).

60. J. P. Zehr, Nitrogen fixation by marine cyanobacteria. *Trends Microbiol.* **19**, 162–173 (2011).

61. L. W. von Friesen, M. L. Paulsen, O. Müller, F. Gründger, L. Riemann, Glacial meltwater and seasonality influence community composition of diazotrophs in Arctic coastal and open waters. *FEMS Microbiol. Ecol.* **99**, fiad067 (2023).

62. L. W. von Friesen, L. Riemann, Nitrogen fixation in a changing Arctic Ocean: An overlooked source of nitrogen? *Front. Microbiol.* **11**, 596426 (2020).

63. C. Barria, M. Malecki, C. M. Arraiano, Bacterial adaptation to cold. *Microbiology* **159**, 2437–2443 (2013).

64. W. Llovel, J. K. Willis, F. W. Landerer, I. Fukumori, Deep-ocean contribution to sea level and energy budget not detectable over the past decade. *Nat. Clim. Chang.* **4**, 1031–1035 (2014).

65. G. C. Hays, A. J. Richardson, C. Robinson, Climate change and marine plankton. *Trends Ecol. Evol.* **20**, 337–344 (2005).

66. P. G. Falkowski, M. J. Oliver, Mix and match: How climate selects phytoplankton. *Nat. Rev. Microbiol.* **5**, 813–819 (2007).

67. Y. Zhou, H. Gong, F. Zhou, Responses of horizontally expanding oceanic oxygen minimum zones to climate change based on observations. *Geophys. Res. Lett.* **49**, e2022GL097724 (2022).

68. S. Chakraborty, K. H. Andersen, A. W. Visser, K. Inomura, M. J. Follows, L. Riemann, Quantifying nitrogen fixation by heterotrophic bacteria in sinking marine particles. *Nat. Commun.* **12**, 4085 (2021).

69. M. Maun, *The Biology of Coastal Sand Dunes* (Oxford Univ. Press, Oxford, 2009).

70. S. J. Pirt, Maintenance energy: A general model for energy-limited and energy-sufficient growth. *Arch. Microbiol.* **133**, 300–302 (1982).

71. K. A. S. Mislan, C. A. Stock, J. P. Dunne, J. L. Sarmiento, Group behavior among model bacteria influences particulate carbon remineralization depths. *J. Mar. Res.* **72**, 183–218 (2014).

72. H. Dalton, J. R. Postgate, Effect of oxygen on growth of azotobacter chroococcum in batch and continuous cultures. *J. Gen. Microbiol.* **54**, 463–473 (1968).

73. Y. Li, L.-L. Sun, Y.-Y. Sun, Q.-Q. Cha, C.-Y. Li, D.-L. Zhao, X.-Y. Song, M. Wang, A. McMinn, X.-L. Chen, Y.-Z. Zhang, Q.-L. Qin, Extracellular enzyme activity and its implications for organic matter cycling in northern chinese marginal seas. *Front. Microbiol.* **10**, 2137 (2019).

74. C. Serra-Pompei, G. I. Hagstrom, A. W. Visser, K. H. Andersen, Resource limitation determines temperature response of unicellular plankton communities. *Limnol. Oceanogr.* **64**, 1627–1640 (2019).

75. R. W. Eppley, Temperature and phytoplankton growth in the sea. *Fish. Bull.* **70**, 1063–1085 (1972).

76. C. A. Durkin, M. L. Estapa, K. O. Buesseler, Observations of carbon export by small sinking particles in the upper mesopelagic. *Mar. Chem.* **175**, 72–81 (2015).

77. J. J. P. Karlusich, E. Pelletier, F. Lombard, M. Carsique, E. Dvorak, S. Colin, M. Picheral, F. M. Cornejo-Castillo, S. G. Acinas, R. Pepperkok, E. Karsenti, C. de Vargas, P. Wincker, C. Bowler, R. A. Foster, Global distribution patterns of marine nitrogen-fixers by imaging and molecular methods. *Nat. Commun.* **12**, 4160 (2021).

78. M. Simon, A. L. Alldredge, F. Azam, Bacterial carbon dynamics on marine snow. *Mar. Ecol. Prog. Ser.* **65**, 205–211 (1990).

79. I. Azúa, M. Unanue, B. Ayo, I. Artolozaga, J. Iriberri, Influence of age of aggregates and prokaryotic abundance on glucose and leucine uptake by heterotrophic marine prokaryotes. *Int. Microbiol.* **10**, 13–18 (2007).

80. P. Lopez-Fernandez, S. Bianchelli, A. Pusceddu, A. Calafat, A. Sanchez-Vidal, R. Danovaro, Bioavailability of sinking organic matter in the Blanes canyon and the adjacent open slope (NW Mediterranean Sea). *Biogeosciences* **10**, 3405–3420 (2013).

81. L. Guidi, G. A. Jackson, L. Stemmann, J. C. Miquel, M. Picheral, G. Gorsky, Relationship between particle size distribution and flux in the mesopelagic zone. *Deep. Res. Part I Oceanogr. Res. Pap.* **55**, 1364–1374 (2008).

82. Z. Wang, S. Hu, Q. Li, H. Liu, G. Wu, Variability of marine particle size distributions and the correlations with inherent optical properties in the coastal waters of the Northern South China Sea. *Remote Sens.* **14**, 2881 (2022).

83. T. Devries, J. H. Liang, C. Deutsch, A mechanistic particle flux model applied to the oceanic phosphorus cycle. *Biogeosciences* **11**, 5381–5398 (2014).

84. R. Kiko, M. Picheral, D. Antoine, M. Babin, L. Berline, T. Biard, E. Boss, P. Brandt, F. Carlotti, S. Christiansen, L. Coppola, L. De Cruz, E. Diamond-riquier, X. D. De Madron, A. Elineau, J. Karstensen, D. Kim, R. M. Lekanoff, F. Lombard, R. M. Lopes, A global marine particle size distribution dataset obtained with the Underwater Vision Profiler 5. *Earth Syst. Sci. Data Discuss.* **14**, 4315–4337 (2022).

85. D. Bianchi, T. S. Weber, R. Kiko, C. Deutsch, Global niche of marine anaerobic metabolisms expanded by particle microenvironments. *Nat. Geosci.* **11**, 263–268 (2018).

86. F. A. C. Le Moigne, C. Cisternas-Novoa, J. Piontek, M. Maßmig, A. Engel, On the effect of low oxygen concentrations on bacterial degradation of sinking particles. *Sci. Rep.* **7**, 16722 (2017).

87. I. Klawonn, S. Bonaglia, V. Brüchert, H. Ploug, Aerobic and anaerobic nitrogen transformation processes in N_2-fixing cyanobacterial aggregates. *ISME J.* **9**, 1456–1466 (2015).

88. D. Boeuf, B. R. Edwards, J. M. Eppley, S. K. Hu, K. E. Poff, A. E. Romano, D. A. Caron, D. M. Karl, E. F. DeLong, Biological composition and microbial dynamics of sinking particulate organic matter at abyssal depths in the oligotrophic open ocean. *Proc. Natl. Acad. Sci. U.S.A.* **116**, 11824–11832 (2019).

89. C. L. M. Steenbergen, H. J. Korthals, M. van Nes, Ecological observations on phototrophic sulfur bacteria and the role of these bacteria in the sulfur cycle of monomictic Lake Vechten (The Netherlands). *Acta Acad. Abo.* **47**, 97–115 (1987).

90. R. F. Vaccaro, S. E. Hicks, H. W. Jannasch, F. G. Carey, The occurrence and role of glucose in seawater. *Limnol. Oceanogr.* **13**, 356–360 (1968).

91. C. Lee, J. L. Bada, Dissolved amino acids in the equatorial Pacific, the Sargasso Sea, and Biscayne Bay. *Limnol. Oceanogr.* **22**, 502–510 (1977).

92. J. Wright, A. Colling, "The seawater solution" in *Seawater: Its Composition, Properties and Behaviour* (Elsevier, ed. 2nd, 1995), pp. 85–127.

93. G. Billen, S. Becquevort, Phytoplankton-bacteria relationship in the Antarctic marine ecosystem. *Polar Res.* **10**, 245–254 (1991).

94. E. Fouilland, M. Gosselin, R. B. Rivkin, C. Vasseur, B. Mostajir, Nitrogen uptake by heterotrophic bacteria and phytoplankton in Arctic surface waters. *J. Plankton Res.* **29**, 369–376 (2007).

95. T. Treude, J. Niggemann, J. Kallmeyer, P. Wintersteller, C. J. Schubert, A. Boetius, B. B. Jørgensen, Anaerobic oxidation of methane and sulfate reduction along the Chilean continental margin. *Geochim. Cosmochim. Acta* **69**, 2767–2779 (2005).

96. R. Kondo, D. B. Nedwell, K. J. Purdy, S. de Queiroz Silva, Detection and enumeration of sulphate-reducing bacteria in estuarine sediments by competitive PCR. *Geomicrobiol. J.* **21**, 145–157 (2004).

97. M. Bentzon-Tilia, I. Severin, L. H. Hansen, L. Riemann, Genomics and ecophysiology of heterotrophic nitrogen-fixing bacteria isolated from estuarine surface water. *MBio* **6**, e00929 (2015).

98. K. J. Flynn, "Incorporating plankton respiration in models of aquatic ecosystem function" in *Respiration in Aquatic Ecosystems*, P. A. del Giorgio, P. J. Williams, Eds. (Oxford Univ. Press, 2005), pp. 248–266.

99. H. Ploug, H. P. Grossart, F. Azam, B. B. Jørgensen, Photosynthesis, respiration, and carbon turnover in sinking marine snow from surface waters of Southern California Bight: Implications for the carbon cycle in the ocean. *Mar. Ecol. Prog. Ser.* **179**, 1–11 (1999).

100. A. Paulmier, I. Kriest, A. Oschlies, Stoichiometries of remineralisation and denitrification in global biogeochemical ocean models. *Biogeosciences* **6**, 923–935 (2009).

101. M. Henze, M. C. M. van Loosdrecht, G. A. Ekama, D. Brdjanovic, *Biological Wastewater Treatment Principles, Modelling and Design* (IWA Publishing, 2008).

102. H. Ploug, U. Passow, Direct measurement of diffusivity within diatom aggregates containing transparent exopolymer particles. *Limnol. Oceanogr.* **52**, 1–6 (2007).

103. M. McCabe, T. C. Laurent, Diffusion of oxygen, nitrogen and water in hyaluronate solutions. *Biochim. Biophys. Acta* **399**, 131–138 (1975).

104. L. Yuan-Hui, S. Gregory, Diffusion of ions in sea water and in deep-sea sediments. *Geochim. Cosmochim. Acta* **38**, 703–714 (1974).

105. W. D. Stein, *Channels, Carriers, and Pumps: An Introduction to Membrane Transport* (Academic Press, 1990).

106. L. Prescott, J. Harley, D. Klein, "Procaryotic cell structure and function" in *Microbiology* (McGrawn Hill, ed. 5th, 2002), pp. 41–73.

107. S. Lee, J. A. Fuhrman, Relationships between biovolume and biomass of naturally derived marine bacterioplankton. *Appl. Environ. Microbiol.* **53**, 1298–1303 (1987).
